# Low‐beta repetitive transcranial magnetic stimulation to human dorsolateral prefrontal cortex during object recognition memory sample presentation, at a task‐related frequency observed in local field potentials in homologous macaque cortex, impairs subsequent recollection but not familiarity

**DOI:** 10.1111/ejn.15535

**Published:** 2021-12-02

**Authors:** Zhemeng Wu, Martina Kavanova, Lydia Hickman, Erica A. Boschin, Juan M. Galeazzi, Lennart Verhagen, Matthew Ainsworth, Carlos Pedreira, Mark J. Buckley

**Affiliations:** ^1^ Department of Experimental Psychology University of Oxford Oxford UK; ^2^ Department of Psychology University of Toronto Scarborough Toronto ON Canada; ^3^ School of Psychology University of Birmingham Birmingham UK; ^4^ Donders Institute for Brain, Cognition and Behaviour Radboud University Nijmegen Nijmegen 6525 XZ the Netherlands

**Keywords:** prefrontal cortex, primate, recognition memory, recollection, rTMS

## Abstract

According to dual‐process signal‐detection (DPSD) theories, short‐ and long‐term recognition memory draws upon both familiarity and recollection. It remains unclear how primate prefrontal cortex (PFC) contributes to these processes, but frequency‐specific neuronal activities are considered to play a key role. In Experiment 1, nonhuman primate (NHP) local field potential (LFP) electrophysiological recordings in macaque left dorsolateral PFC (dlPFC) revealed performance‐related differences in a low‐beta frequency range during the sample presentation phase of a visual object recognition memory task. Experiment 2 employed a similar task in humans and targeted left dlPFC (and vertex as a control) with repetitive transcranial magnetic stimulation (rTMS) at 12.5 Hz during occasional sample presentations. This low‐beta frequency rTMS to dlPFC decreased DPSD derived indices of recollection, but not familiarity, in subsequent memory tests of the targeted samples after short delays. The same number of rTMS pulses over the same total duration albeit at a random frequency had no effect on either recollection or familiarity. Neither stimulation protocols had any causal effect upon behaviour when targeted to the control site (vertex). In this study, our hypotheses for our human TMS study were derived from our observations in NHPs; this approach might inspire further translational research through investigation of homologous brain regions and tasks across species using similar neuroscientific methodologies to advance the neural mechanism of recognition memory in primates.

## Graphical abstract

Frequency‐specific neural activities play essential roles in recognition memory, which is deemed to draw upon familiarity and recollection. In our study by first recording macaque local field potentials (LFPs), we observed modulation of low‐beta frequency activity in dorsolateral prefrontal cortex (PFC) during sample stimulus encoding, which was implicated in task performance. Then, in an analogous recognition memory task in humans, we targeted the homologous brain region (i.e., left dorsolateral PFC) with repetitive transcranial magnetic stimulation (rTMS) at low‐beta frequency and succeeded in causally influencing mnemonic function during encoding of targeted samples. We revealed an effect of low‐beta frequency stimulation to dorsolateral PFC in suppressing recollection process rather than familiarity process. These cross‐species comparison studies demonstrate a causal role of low‐beta frequency activities in PFC to primate recognition memory performance.

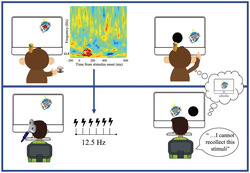

**Abbreviations**
BBA: beta band activity
dlPFC: dorsolateral prefrontal cortex
DPSD: dual‐process signal detection
EEG: electroencephalogram
ITIs: intertrial intervals
LFP: local field potential
MEG: magnetoencephalogram
MRI: magnetic response imaging
MTL: medial temporal lobe
NHP: nonhuman primate
PFC: prefrontal cortex
RMT: resting motor threshold
ROC: receiving‐operating characteristic
rTMS: repetitive transcranial magnetic stimulation

## 1 INTRODUCTION

A significant body of research maintains that human recognition memory draws upon both familiarity and recollection, according to dual‐process theory (Cipolotti et al., 2006; Wixted, 2007; Yonelinas, 1994, 2001; Yonelinas et al., 1998, 2002; Yonelinas & Parks, 2007). Familiarity is defined as a general sense of knowing the item/object was encountered before but independent of the contextual environment; whereas recollection is characterized by vivid recall of the contextual information about the episode in which the item/object was previously encountered (Scalici et al., 2017; Wixted, 2007; Yonelinas, 2001, 2002; Yonelinas & Parks, 2007). According to dual‐process signal‐detection (DPSD) models, familiarity is thought to be a signal‐detection process that generates memories of varying confidence levels, whereas recollection is considered to be a threshold process that generates high confidence‐level memories (Yonelinas, 2001, 2002). An associated receiver‐operating characteristic (ROC) curve analysis approach plots hits against false alarms across cumulative confidence levels. This ROC/DPSD approach requires participants to report their confidence in the accuracy of their judgements that each stimulus has been viewed before. Accordingly, the ROC approach is markedly easier in humans than in nonhuman animals though there has been some success in the latter too (Fortin et al., 2004; Guderian et al., 2011; Sauvage et al., 2008). Regardless, the typical ROCs that result in nonamnesic subjects have a symmetrical and curvilinear component associated with familiarity and an asymmetrical and linear component related to recollection. Hence, differential contributions of recollection and familiarity to the recognition memory task may be ascertained from the curve's shape; more formally, the recollection and familiarity indices are extracted from curve fitting (Koen et al., 2017; Yonelinas, 2001, 2002; Yonelinas & Parks, 2007). This ROC/DPSD approach is traditionally associated with long‐term memory, but many tasks used in the aforementioned literature assess relatively short‐term memories with this approach (see Section 4).

In the context of prefrontal cortex (PFC) functional investigations, such short‐term recognition memory tasks are often referred to as delayed response or working memory tasks, but there is no consensus as to whether different subregions within PFC contribute differently to familiarity and recollection. Using paradigms that aim to dissociate these processes, human neuropsychological investigations present conflicting results. For example, in some studies, lateral PFC lesions cause deficits more in familiarity rather than recollection (Aly et al., 2011; Duarte et al., 2005; MacPherson et al., 2008), whereas in other studies such lesions did not impair either (Wheeler & Stuss, 2003). Evidence from human neuroimaging studies also provides very mixed evidence as to the relative contributions of PFC subregions to familiarity/recollection (Henson, Rugg, et al., 1999; Henson, Shallice, & Dolan, 1999; Johnson et al., 2013; Kafkas & Montaldi, 2012; Scalici et al., 2017; Skinner & Fernandes, 2007).

A long‐held influential view in the field has been that sustained neuronal spiking in PFC encodes stimuli across delays in short‐term/working memory tasks (see Leavitt et al., 2017 for a review). However, more recently, other candidate mnemonic mechanisms have been proposed. Some of these involve dynamic attractor models wherein the maintenance of working memory is mediated by ‘activity‐silent’ selective synaptic changes. One feature of these models is that periodic neuronal firing/spiking is linked to short‐lived attractor states associated with frequency‐specific powers of local field potential (LFP) activities (Lundqvist et al., 2016; Stokes, 2015). However, relatively few studies to date have analysed LFP activity in nonhuman primate (NHP) lateral PFC during working memory tasks. Fewer studies still have attempted to draw links between electrophysiological investigations of LFP in NHPs and investigation of human performance on similar tasks.

Hence, here we aimed to test a broad proof of principle that LFP parameters (frequency, location, timing) derived from electrophysiological recording methodologies in NHPs might generate testable hypotheses for complementary investigations in humans. To that end, we chose repetitive transcranial magnetic stimulation (rTMS) as an interference methodology in humans that could deliver frequency and location and timing specific stimulation to ascertain if causal links could be found despite species differences and some differences between tasks. It has already been established that rTMS either before or during task performance can modulate ongoing neural oscillations (Huang et al., 2005; Rossi & Rossini, 2004). Moreover, it has previously been observed that behavioural performance in humans can be either disrupted or enhanced by adopting rTMS in a frequency‐specific manner (Albouy et al., 2017; Chanes et al., 2013; Elkin‐Frankston et al., 2011; Thut et al., 2011). For example, high‐beta frequency rTMS over frontal eye fields (an area of human PFC) enhances perceptual discrimination, whereas response criterion is lowered by delivery of rTMS over the same region at a gamma frequency (Chanes et al., 2013). As for the choice of a working memory/short‐term recognition memory task suitable for both primate species, we opted for a well validated task (Wu et al., 2020), itself based on a paradigm introduced by Basile and Hampton (2013). This task has established variants for NHPs and humans that differ to a degree but nonetheless elicit broadly similar performance profiles in human and macaques (Wu et al., 2020). Specifically, both species show corresponding profiles of so‐called fast familiarity (FF) and slow recollection (SR) responses. Moreover, Wu et al. (2020) validated these FF/SR profiles against ROC‐based dual‐process model fitting. That said, debate remains as to whether animals also provide suitable models of recollection (e.g., see Eichenbaum et al., 2005, 2008; Wixted & Squire (2008)), although it has been shown, using ROC procedures and dual‐process models similar to those in humans, that processes akin to recollection exist in macaques (Guderian et al., 2011). These approaches are particularly robust in rodents wherein indices of recollection have additionally been shown to be causally dependent upon an intact hippocampus (Fortin et al., 2004; Sauvage et al., 2008) as is the case in humans. This literature raises the distinct possibility then that direct extracellular recordings in NHPs while they perform recognition memory tasks may provide some clues about the neural mechanisms that underlie recollection and familiarity processes in primates.

Hence, we conducted a two‐part study. First, in Experiment 1, we recorded extracellular recordings in two macaques during performance of the short‐term object recognition memory task to observe what parameters of performance‐related LFP power might exist in dorsolateral PFC (dlPFC) during sample encoding. Second, in Experiment 2, we used those parameters to derive hypotheses as to the causal effects of spatially and temporally targeted rTMS to analogous regions of human PFC.

The first performance‐related LFP observation in Experiment 1 was that power in the 10‐to 15‐Hz band peaked higher in trials that proceeded to be correct (hit) trials in the subsequent choice phase than in trials that proceeded to be incorrect (miss) trials. Heightened beta band activity (BBA) has previously been linked to attention. One magnetoencephalogram (MEG) study showed increased 8‐ to 14‐Hz activity in frontal regions for perceived stimuli but not for unperceived stimuli lasting 30–150 ms after stimulus onset (Palva et al., 2005), perhaps indicating an early involvement of alpha/low‐beta power in visual attention (Palva & Palva, 2011) and stimulus perception (Palva et al., 2005). Hence, in Experiment 2, we used 12.5‐Hz rTMS stimulation (as this frequency was exactly intermediate in the overlapping frequency bands significant in both NHPs in our permutation tests of sample‐related LFP activity in trials destined to be either hits vs. misses) and hypothesized that this low‐beta rTMS during the sample presentation epoch in the human version of the task might enhance attention (via BBA entrainment) resulting in improved memories of those samples targeted at test.

The second performance related LFP observation in the two NHPs in Experiment 1 was that, after the aforementioned BBA peak, the rate of BBA power reduction from that peak was greater in trials that proceeded to be hit trials than the corresponding negative gradient observed in trials that proceeded instead to be miss trials. According to information by desynchronization theory (Hanslmayr et al., 2012), significant post‐discriminanda reduction in BBA is associated with the idea that the richness of information represented in the brain is positively related to alpha/beta power decreases/desynchronization. For example, Hanslmayr et al. (2014) showed that only beta frequency rTMS to human inferior frontal gyrus impaired memory encoding. Moreover, in that study, only beta frequency stimulation led to a sustained oscillatory echo, and the echo outlasted the stimulation period by 1.5 s and had a strength related to memory impairment. According to this theory, the artificial entrainment of a sustained exogenous beta oscillatory rhythm prevented effective desynchronization of normal exogenous beta rhythms accompanying effective encoding. In our Experiment 1, we observed more rapid reduction in BBA in macaque dlPFC after sample presentation in trials that proceeded to be hit trials as opposed to miss trials. Hence, we also formed an alternative hypothesis, namely, that targeting the homologous human region with 12.5‐Hz rTMS during sample presentation may impair human participants' subsequent memories for those samples targeted with rTMS (postulating, similarly, that beta entrainment interferes with optimal desynchronization).

As a control procedure, we also investigated randomly timed rTMS with the same number of pulses as in our beta rTMS and over the same total duration but with no inherent pulse frequency. With respect to our first hypothesis, random rTMS during the sample presentation was not expected to enhance attention to the sample. Indeed, if attention was beta frequency dependent, we would expect random rTMS to reduce attention and impair sample memory. With respect to our second hypothesis, random rTMS was not expected to entrain BBA after sample onset; hence, random rTMS was not expected to delay desynchronization and accordingly not deleteriously affect sample encoding and subsequent recognition. We also hypothesized that rTMS stimulation would affect recollection more than familiarity after Scalici et al.'s (2017) meta‐analyses discussed earlier. Finally, we included vertex as a control stimulation site and accordingly hypothesized no effect of rTMS to vertex on either of these two memory processes, by either of the two rTMS stimulation protocols.

## 2 MATERIALS AND METHODS

### 2.1 Experiment 1: NHP electrophysiological study

#### 2.1.1 Subjects

Neural data were recorded in two young adult macaque monkeys (*Macaca mulatta*, one female aged 8 years, weight 10–13 kg; the other male aged 9 years, weight 11–15 kg). All animals in our lab are socially housed (or socially housed for as long as possible if later precluded, e.g., by repeated fighting with cage mates despite multiple regrouping attempts), and all are housed in enriched environments (e.g., swings and ropes and objects, all within large pens with multiple wooden ledges at many levels) with a 12‐h light/dark cycle. The NHPs always had ad libitum water access 7 days/week. Most of their daily food ration of wet mash and fruit and nuts and other treats were delivered in the automated testing/lunch box at the end of each behavioural session. This provided ‘jackpot’ motivation for quickly completing successful session performance. This was also supplemented by trial‐by‐trial rewards for correct choices in the form of drops of smoothie delivered via a sipping tube. Additional fruit and foraging mix was provided in the home enclosure. All animal training and procedures were performed in accordance with the guidelines of the UK Animals (Scientific Procedures) Act of 1986, licensed by the UK Home Office, and approved by Oxford's Committee on Animal Care and Ethical Review.

#### 2.1.2 Surgical procedures

After the initial task training phase was complete, both animals had head‐post implantation surgery. Monkey A received a titanium head‐post, and Monkey B received a custom‐designed PEEK head‐post (a magnetic response imaging [MRI] compatible implant). Both head‐posts were implanted posteriorly and secured to the cranium with titanium cranial screws through the legs of the post shaped to fit the precise morphology of the skull in the region according to a 3D‐printed skull models based on preoperative structural MRI scans. After implantation, the reflected skin and galea (themselves cut in a way to minimize interruption to blood supply) were sutured, and the wound closed around the base of the head‐post in layers so that, finally, only the narrow diameter post protruded through the skin.

Later, after behavioural training with head‐fixation was complete and task‐performance satisfactory, both NHPs received surgical implantation of electrodes. Monkey A had implantations of microelectrode arrays (Utah arrays, Blackrock Microsystems). During the array implantation surgery, a bone flap was raised over the left anterior PFC, the dura mater was cut and reflected, and Utah arrays implanted directly into the cortex (including dlPFC) with the aid of an operating microscope for intracranial surgeries. The dura mater was sewn back over the arrays where possible, and the bone flap was replaced and sewn through small holes. Two reference wires connecting to the pedestals were left under the dura away from the site of the arrays, and these and the wire bundle connecting to each electrode in the Utah array exited the cranium through a gap at the edge of the craniotomy and ran from there to the pedestal that was secured to the cranium (located away from the edge of the craniotomy) with titanium cranial screws. To complete the procedure, the wound was then closed in layers. A pedestal cap was screwed on to the top of the pedestal to protect the gold contacts (the cap was subsequently removed each day, while the NHP in the recording session, to connect the digital head‐stage direct to the pedestal's connectors at which point recording commenced from the chronically implanted electrodes).

In Monkey B, we targeted multiple electrodes to dlPFC using a different method. Monkey B received implantations of chamber and microdrive system (Gray Matter Research). The bespoke form‐fitting cranial chambers (and their internal multielectrode microdrive system) were designed to fit the animal by creating an MRI‐based 3D model of the skull using Brainsight software (https://www.rogue-research.com/tms/brainsight-tms/). Targeted brain regions were outlined in 3D Slicer software (https://www.slicer.org) and chamber trajectories planned accordingly. In this monkey, we aimed one chamber at left PFC and one chamber at left medial temporal lobe (MTL); the latter of which was incorporated for another study. The implantation surgeries for each chambers/microdrive system were conducted separately. Prior to chamber implantation surgeries, MRI‐based skull models with stereotaxic coordinates were initially marked by a point, and each chamber outline was marked by a circle. In this step, the stereotaxic coordinates of the centre of each chamber's location were recorded with respect to ear‐bar origin (i.e., PFC chamber: anterior–posterior = +26.5 mm, medio‐lateral = +22.0 mm, dorsal–ventral = +10.0 mm; MTL chamber: anterior–posterior = +6.0 mm, medio‐lateral = +15.0 mm, dorsal–ventral = +30.0 mm). During chamber implantation surgeries, once the chamber locations were identified in a stereotaxic frame, several holes around the chamber were drilled, and titanium bone screws were then anchored on the skull. Based on the recorded locations in the stereotaxic system, chambers were next positioned using the stereotaxic chamber holder mounted onto a coarse manipulator and then were sealed by a thin bead of superbond bone cement. Once the sealing was complete, a thin layer of bone cement was applied to connect the chambers to the surrounding bone screws, and the stereotaxic chamber holder was removed. Additional layers of bone cement were built up to complete the coverage of the screws. Finally, the o‐ring, short plug and chamber cap were placed in order to ensure the chamber interior could be kept as sterile as possible.

After ~2 months, Monkey B received the microdrive implantation surgery. During this implantation surgery, a craniotomy was made within the interior of the chambers. A starter hole was made in the centre of the chamber using a bone drill, and then all of the remaining bones were removed using a rongeur. The dura beneath the removed bones was then punctured by the grid using a sterile needle, to guarantee in the later stage that the microdrive electrodes could be driven through the dura smoothly so to minimize the possibility of bending or damage to the electrode tips. After dura puncture, the frontal multielectrode microdrive system (Gray Matter Research SC32, inner chamber diameter: 20.30 mm) was mounted within the anterior chamber, and posterior multielectrode microdrive system (Gray Matter Research SC96, inner chamber diameter: 23.37 mm) was mounted within the posterior chamber. The implanted microdrives were fixed in place by tightening the retaining caps onto the chambers, then an additional layer of bone cement was applied to reinforce the microdrive stability. Finally, all the microdrives were fully assembled with protective caps, with fixation screws tightening on the chambers. All electrodes were gradually lowered to the targeted areas (e.g., dlPFC for this study) across days and daily recordings from dlPFC commenced when sufficient electrodes tips were in this target location.

All operations were performed in aseptic conditions. Monkeys were first sedated with ketamine (5 mg/kg), medetomidine (20 mcg/kg) and midazolam (0.1 mg/kg) given i.m., intubated, and then artificially respirated using a mixture of carrier gases (oxygen/medical air) and volatile anaesthetic. Surgical depth of anaesthesia was maintained throughout the surgery with sevoflurane (1.0%–2.0% to effect) and injectable adjuncts (fentanyl 5 mcg/kg/h i.v., dexmedetomidine 0.5 mcg/kg/h i.v.). On average, three doses of steroids (methylprednisolone 20 mg/kg i.v. every 4–6 h) and, in the case of intracranial surgery, a bolus of mannitol (1,000 mg/kg i.v.) were given on the day of surgery to protect against intraoperative brain edema and postoperative inflammation. Steroids were continued in the postoperative phase (dexamethasone 0.2 mg/kg s.c., daily for 5 days). The animals were given an antibiotic (30 mg/kg of amoxicillin intraoperatively every 2 h, and 17.5 mg/kg daily postoperatively) for prophylaxis of infection. Additional intraoperative medication included atropine (10 mcg/kg i.v. as required), an H2 receptor antagonist ranitidine (1 mg/kg i.v.), meloxicam (0.2 mg/kg i.v.) and crystalloid fluids (Hartmann's solution 3–5 ml/kg/h). Heart rate, oxygen saturation of haemoglobin, mean arterial blood pressure, end‐tidal CO_2_, body temperature and respiration rate were monitored continuously throughout surgery. Postoperative analgesia was provided via opioids (methadone 0.3 mg/kg i.m. or buprenorphine 10 mcg/kg i.m.) and nonsteroidal anti‐inflammatory agents (0.1 mg/kg of meloxicam, p.o./i.m., 10 mg/kg acetaminophen p.o.). A proton pump inhibitor (omeprazole 0.5 mg/kg) was given daily to protect against gastric ulceration as a side effect of the combination of steroid and nonsteroidal anti‐inflammatory treatment.

**FIGURE 1:**
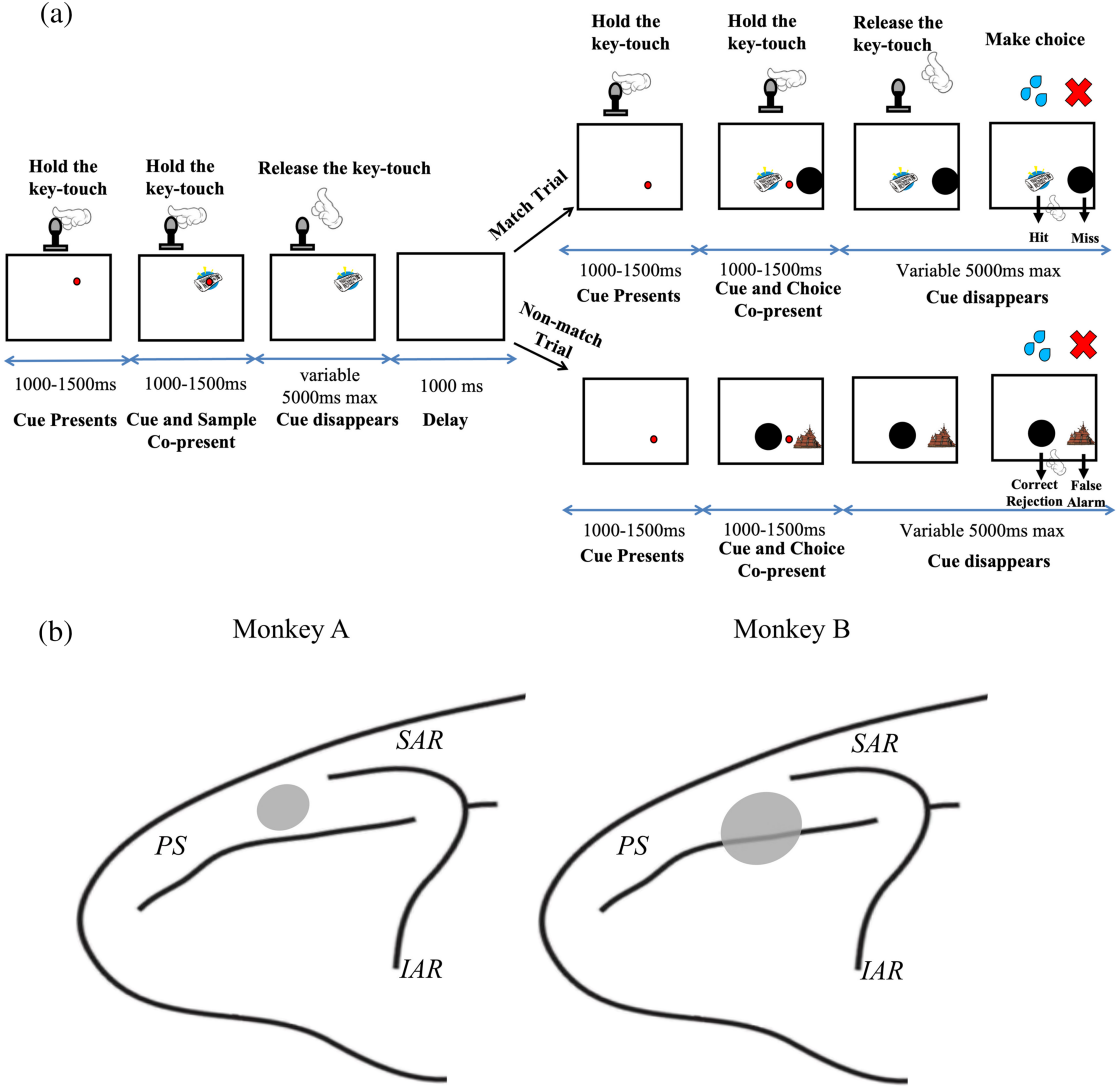
Nonhuman primate (NHP) recognition memory task structure and recording sites from dlPFC in two NHPs. Panel (a) depicts the structure of one trial in the electrophysiological study in Experiment 1. The NHPs initiated the task by holding the key touch device once the red dot cue appeared, after which a sample object image was shown on the screen behind the red dot cue (encoding phase). The NHPs were required to keep holding the key touch device after which the red key touch cue disappeared, then the NHPs were trained to release their hands from the key touch prompting the commence of the delay (1,000 ms) between sample and choice phases. Then, after the delay period another key touch red dot cue appeared, animals were required to hold the key touch again until the red cue disappeared. After the two choice stimuli (one an object image and the other a black circle) appeared, animals were trained to make a choice to the touchscreen to either the object test‐image stimulus or to the black circle. Just as in the human task in Figure 2 for Experiment 2, in ‘match trials’, the black circle was presented with an identical image to one of the preceding sample, whereas in ‘nonmatch trials’, the black circle was presented with a novel stimulus not seen before. Animals were trained to touch the test item if they remembered the test item was a match but to touch the black circle if they thought the test item was a nonmatch; accordingly, the responses could be separated into hits, misses, correct rejections and false alarms as indicted. Panel (b): recording locations in macaque lateral prefrontal cortex (PFC); both recording sites were anterior to the superior arcuate sulcus (SAR) and inferior arcuate sulcus (IAR), located above the principal sulcus (PS) in Monkey A and around the PS in Monkey B, which were marked by grey areas

This study records data from one 32‐electrode Utah array inserted into dlPFC (area 9/46, just dorsal to the principal sulcus, see Figure 1b) in Monkey A and from one 32‐electrode microdrive advanced into lateral PFC (see Figure 1b) in Monkey B. The Utah array electrodes in Animal A were made of platinum (Pt), arranged on a 6 × 6 grid embedded in silicone; the electrode length was 1.0 mm with interelectrode spacing of 0.4 mm. The microdrive electrodes in Animal B were a mixture of 10 Platinum/Iridium (Microprobes, USA) and 22 tungsten microelectrodes (Alpha Omega, USA). These microdrive electrodes had a 1.5‐mm spacing between adjacent locations, and each was independently controlled in depth by a microdrive.

#### 2.1.3 Task stimuli and apparatus

The object recognition memory task we used was programmed using Turbo Pascal (Borland) and run under DOS on a desktop PC. Object stimuli used in the task were multicoloured clip‐art images presented on a 20.1″ colour touch‐sensitive screen (TFT LCD TS200H GNR). Clip‐art images used in the task were drawn from a large pool of several thousand unique images. Each image subtended 5° of visual angle in width and 5° in height to the subject when presented on the screen. The sample stimulus was always presented on the right top of the screen, positioned +9° horizontal and −9° vertical from the centre of the screen. The test stimuli were presented on the right bottom of the screen: One was positioned 0° horizontal and +3° vertical from the centre of the screen; and the other one was positioned +17° horizontal and +3° vertical from the centre of the screen. The background colour to the screen was white. In each session of recordings, images were randomly chosen from the pool without replacement and were not re‐used on the other recording days.

The animals were seated in a primate chair (Rogue Research Inc.) in front of the touch screen with its head‐fixated while they performed the recognition memory task in a partially magnetic‐shielded, and partially sound‐attended, testing box. A window in the front of the chair provided their access, both to the touchscreen itself and also to a touch‐sensitive knob which we refer to as a ‘key touch’, which was positioned low down in front of the touch screen. Animals had to steadily hold the key touch at various times in each trial. This was to control for arm movement/position while they waited for stimuli and looked at stimuli. The distance between the two monkeys and touch screen was fixed at 50 and 22 cm, respectively, enabling them to touch the screen easily. The distance was adjusted between monkeys to make sure their right hands could reach both the key touch and the screen. An infrared camera was used to monitor the general status of the monkeys in the box. A peristaltic pump device located on top of the box fed smoothie reward through a tube and to a spout positioned in the vicinity of the animals' mouths. Below the screen was also an automated lunch box that contained the majority of the animals' daily meal (wet mash and fruits and nuts etc.) and that opened immediately at the end of the task to provide the ‘jackpot’ end‐of‐task motivator.

#### 2.1.4 Behavioural task

The task is a variant of the well‐established delayed matching‐to‐sample recognition memory paradigm in which an object stimulus in a sample phase has to be judged as familiar or not in a subsequent choice phase after a short delay. The form of the task we used is similar in broad terms to that used by Basile and Hampton (2013) and very similar indeed to the version we created and used in an earlier study (Wu et al., 2020). In all these tasks, a key task design feature is that in the choice phase, to allow separation of hits, misses, false alarms, and correct rejections, one choice stimulus is presented along with one nonmatch button (black circle) such that the animals have a binary choice. They should select the choice stimulus if they consider the choice stimulus a match to the previously seen sample; else select the black circle if they consider it is a nonmatch (see Figure 1a). In each trial, the animals initiated the trial by holding the key touch when cued to do so by a small red circular key touch cue (located towards upper centre of screen; Figure 1a) presented on the screen. The NHPs were then required to keep holding the key touch device through a variable delay of 1,000–1,500 ms (if the key touch hold was broken, the trial aborted) after which a central sample (object stimulus) appeared behind the red circular key touch cue. The NHPs were again required to keep holding the key touch device through another variable delay of 1,000–1,500 ms (again, if the key touch hold was broken, the trial aborted) after which the red key touch cue finally disappeared. The removal of the red circle cued to the animals that they could release their hands from the key touch at which point the delay (1,000 ms) between sample and choice phases began. The maximal time for releasing key touch was 5,000 ms; else the trial aborted, and the animals received a time out for 10 s. Then, after the 1,000‐ms delay period, another red circular key touch cue appeared, this time in the bottom of the screen (close to, and equidistant between, where the two choice items would appear), and animals were required to hold the key touch again. The NHPs were again required to keep holding the key touch device through a variable delay of 1,000–1,500 ms (if the key touch hold was broken, the trial aborted) after which the two choice stimuli appeared, one an object stimulus and the other a black circle as describe above (these two ‘choices’ were left–right randomized between trials). The NHPs were similarly required to keep holding the key touch device through another variable delay of 1,000–1,500 ms (and again, if the key touch hold was broken, the trial aborted) after which the red key touch cue disappeared, which was the cue to the animals that they could release holding the key touch and make a choice to the touchscreen to either the object test stimulus or to the black circle stimulus. The aim of requiring animals to hold the key touch was to minimize their hand movements when viewing the sample or choice images to avoid movement‐related noise during the recording. However, by this approach, their true response time for making their free choice in the choice phase of the task could not be accurately recorded. This is the reason that response time was not involved in our analyses in this study. However, our previous behavioural study analysed response times in detail in a close variant of the same task (Wu et al., 2020). The object stimulus in the choice stage was either the identical stimulus to the sample seen earlier in the trial, or it was not identical to the sample. The animals were rewarded by delivery of 10 ml of smoothie for touching the test‐object stimulus if they chose it and if it correctly matched the sample image (in these trials, we refer to as ‘match trials’). Alternatively, the animals were rewarded for selecting the standard ‘nonmatch button’ (i.e., the black circle) if the test‐object stimulus was a nonmatch (in these trials, we refer to an ‘nonmatch trials’). After a correct response, the intertrial interval (ITI) was 3 s. However, following any error trial (including both incorrect response and aborted trials), the intertrial interval was 10 s in order to encourage good performance. Accordingly, on *match trials*, the animals could either make a correct response (‘hit’) or an incorrect response (‘miss’), whereas on *nonmatch trials*, the animals could either make a correct response (‘correct rejection’) or an incorrect response (‘false alarm’). In this way, the paradigm is similar in design to that used by Basile and Hampton (2013) with a key difference being we did not restrict the stimulus set to just two stimuli as they did; rather, we used larger stimulus sets. Moreover, we introduced a new task element and varied the degree to which stimuli were either familiar or novel in the session. Specifically, in any given session, 50% of the trials used trial‐unique stimuli, and 50% were ‘repeat’ stimuli used previously in the session (but not used in any previous session). Individual sessions were structured such that there were six ‘repeat’ stimuli (composed of three pairs of stimuli) and in each ‘repeat’ trial, one of the three pairs was chosen at random and one member of the pair was randomly chosen to be the sample for that repeat trial. Each session typically consisted of 150 trials, so the six repeat stimuli got steadily more familiar across the session. The repeat trials and trial‐unique stimulus trials were randomly intermixed throughout the session.

#### 2.1.5 Electrophysiological recordings

Data were recorded simultaneously from 32 dlPFC electrodes in both monkeys throughout the recognition memory task. Both NHPs had learnt the task in full prior to implantation surgeries, and both performed the task with a sufficiently high level of accuracy. In this study, we only considered the trial‐unique stimuli in our analyses as we only used trial‐unique stimuli in our human TMS study in Experiment 2. Monkey A accrued, on average, 35 trial‐unique stimulus trials per day. The average session duration was 45 min. The data were recorded over 29 sessions approximately 8–12 weeks after the array implantation. Neural signals from each microelectrode were amplified, digitized at 30 kHz using a 256‐channel Cerebus™ Neural Signal Processor (Blackrock Microsystems). Monkey B accrued, on average, 80 trial‐unique stimulus trials per day. The average session duration was 80 min. The data were recorded over eight sessions approximately 2 weeks after the microdrive implantation. We recorded neural activities simultaneously from 11 electrodes inside dlPFC in Monkey B in this study (the other prefrontal electrodes were advanced to other prefrontal regions for the purpose of another study). Neural signals from each microelectrode were amplified and digitized at 25 kHz using a 256‐channel Ephys Neural Signal Processor (Tucker Davis Technologies). The LFPs for both monkeys were analysed offline using the FieldTrip toolbox (http://www.fieldtriptoolbox.org; for details, see Oostenveld et al., 2011) and using a custom MATLAB script (available upon request from corresponding author).

#### 2.1.6 Signal analysis

We investigated the power of the induced LFP responses during the sample presentation and encoding phase of the NHP recognition memory task. A notch filter, the second‐order infinite impulse response, was applied to the raw data signals, to remove wall power noise (i.e., 50 Hz) and its harmonics (i.e., 100 and 150 Hz). Then, the data signals were down sampled to 1 kHz in both monkeys. To obtain the power spectrums of LFPs for each of the recorded channels, a time‐frequency decomposition analysis based on Morlet wavelet transform was performed. The frequency range in LFP spectral analysis ranged from 8 to 60 Hz (see below for justification), in steps of 1 Hz.

For each electrode channel (i.e., 32 channels in Monkey A and 11 channels in Monkey B), the induced power spectra from LFPs were limited to data segments that contained completed match trials and associated intertrial intervals (ITIs). In each trial, the duration of sample image presentation was 1,000–1,500 ms, while the NHPs held the key touch throughout to control for hand movements. Our analyses of LFPs considered a time window 1,000 ms before stimulus onset until 1,000 ms after stimulus onset for analysis. A 1,000‐ms segment during the ITI was picked as the baseline for all the power spectra, and their root mean square was divided from the raw data before the calculation of the spectrum using a wavelet transform. In the recognition memory task, LFPs in both the pre‐ and post‐sample presentation periods (both within a time window of 1,000 ms while the NHPs held the key touch) from completed hit trials (*N* = 346 in Monkey A; *N* = 330 in Monkey B) and completed miss trials (*N* = 118 in Monkey A; *N* = 8 in Monkey B) were selected and analysed. To obtain the gradient for the alpha/lower beta band (10–15 Hz), we took the LFP data between 100 and 250 ms in Monkey A and between 150 and 300 ms in Monkey B and performed a linear fit to each of the frequencies and to each of the monkeys separately (using the polyfit function in MATLAB).

#### 2.1.7 Statistical methods

To test for statistical significance of differences of the induced LFP power spectra between pre‐ and post‐sample presentations during the recognition memory task, we performed a nonparametric permutation test, with the median difference between the above two conditions (i.e., pre‐ and post‐sample) as our test statistic. The nonparametric permutation test is an assumption‐free method without prescribing underlying distributions (Nichols & Holmes, 2001). In each trial, the time window of pre‐ and post‐sample conditions for nonparametric permutation test was 800 ms (i.e., time zero was the stimulus onset), to avoid the spectral leakage of LFP power when calculated near the margin of a 1,000‐ms time window. An aim of this analysis in Experiment 1 was to determine a suitable stimulation frequency. Hence, we opted not to consider frequencies below 8 Hz because rTMS stimulation at such low frequencies, in the alpha/theta range, would not allow sufficient pulses to accrue per sample presentation (given its duration of only 480 ms) so to set up a ‘frequency’ and also a distinct random control pulse train. We opted to not consider rTMS above 60 Hz due to safety concerns of sustained and repetitive high frequency stimulation (Rossi et al., 2009). Hence, the frequency range of interest for this particular analysis was set to range between 8 and 60 Hz. The nonparametric permutation test was performed separately for hit trials and miss trials for each monkey. In each trial type, the null hypothesis was that there was no significant difference of modulation of LFP power when comparing the observed difference between the two conditions (i.e., pre‐ and post‐sample) against a reference distribution of differences between the two randomly assigned conditions. For each frequency and each time point, the reference distribution was obtained by performing 10,000 permutations on the trial labels to randomly assign them to two ‘conditions’. Then, accordingly, on each loop of the permutation, the median LFP power of that frequency and that time point (for each of the two randomly constituted/permuted ‘conditions’) was calculated, and only the minimal and maximal difference was stored. This resulted in both a minimal and maximal matrix of LFP power, each 53 by 800 (i.e., frequency points × time points). The upper and lower thresholds were defined as the 97.5th percentile of the maximal matrix and the 2.5th percentile of the minimal matrix, respectively. Any observed median difference between pre‐ and post‐sample conditions, greater than the upper threshold or smaller than the lower threshold, was declared significant at the .05 level (*p* < .05, two‐tailed). By selecting the maximal/minimal value from the permutation distribution, this two‐sided nonparametric permutation‐based test was sensitive to both positive and negative changes in LFP power spectra and therefore controlled for global type I errors associated with multiple comparisons (Nichols & Holmes, 2001).

### 2.2 Experiment 2: Human TMS study

#### 2.2.1 Participants

Eighteen participants (12 male, 6 female, age range 18–30 years) took part in the TMS study. Participants were fluent English speakers, right‐handed and had normal or corrected‐to‐normal vision. Prior to the study, all the participants provided written consent and went through safety screening check to make sure they had no history of previous or current neurological or psychiatric conditions and were not taking any psychoactive medication. All the participants received monetary compensation for their participation at a standard rate for volunteers for TMS studies in Oxford. This study was carried out with the approval of Medical Sciences Interdivisional Research Ethics Committee, University of Oxford (R50367/RE001). These participants were tested as part of the final year undergraduate research projects of the then student co‐authors on this study (and as such the total participants recruited had a practical upper limit).

#### 2.2.2 Stimulation brain sites

In this study, we investigated the differential effects of rTMS to dlPFC in the performance of a recognition memory task similar to that used with NHPs in Experiment 1. We chose to stimulate left dlPFC consistently which approximately corresponded to area BA46 or BA9/46 (referred to as BA 9/46) in light of Experiment 1, which also recorded from left dlPFC in both NHPs (but this study is not able to explore issues of laterality). A control brain site was chosen, namely, vertex, which we had no reason to expect to be important for either recollection or familiarity memory process, and which was localized at centre point of the head. The two targeted sites were measured using Beam F3 localization system (Beam et al., 2009). This system allows the measurement of the location of the F3 electrode position in the 10–20 electroencephalogram (EEG) coordinate system, and takes into account individual skull variations. Based on this system, BA 9/46 was localized 1 cm caudal to F3; and vertex was localized at a site corresponding to location of electrode Cz (measured as half the distance between inion and nasion and intersecting with half the distance between the two aural tragi). The relative positions of two stimulation sites are illustrated in Figure 4a. The participants were assigned to one of the two brain site groups to equalize numbers per group: dlPFC group (BA 9/46, 9 participants) and control group (9 participants).

#### 2.2.3 Defining participant's motor threshold

Prior to the experiment, all the participants were invited to a taster session, in which the individual's resting motor threshold (RMT) was obtained for each participant. To measure RMT, stimulation was applied over the site of left primary motor cortex (localized 5 cm laterally and 5 cm rostrally to the vertex), where the largest twitch in right index finger of the participant was found. Site search was initially started at the lowest stimulator output. By gradually increasing output, the RMT of the participant was determined when 5 out of 10 TMS pulses caused a twitch in the right index finger (Rossini et al., 1994).

A stimulation intensity of 90% of RMT was used in the rTMS study. This intensity was within an appropriate range taking into account the average scalp‐cortical surface distance between primary motor cortex and stimulation areas and a 2.8% reduction in stimulator output for every mm closer to the skull (Stokes, 2005; Stokes et al., 2007, 2013). The mean RMT of dlPFC group was 44.33 (SE = 3.61), and of vertex group was 49.11 (SE = 7.39).

#### 2.2.4 Task stimuli and apparatus

The object recognition memory task we used was very similar to the one used in NHPs in Experiment 1 and was similarly programmed using Turbo Pascal (Borland), run under DOS on a desktop PC and presented on a 20.1″ colour touchscreen (TFT LCD TS200H GNR). The object images used in the task were clip‐art images as in Experiment 1 study, but in order to increase difficulty (in light of our pilot study investigations wherein performance of human participants was close to ceiling), the stimuli were all converted to grey scale and the contrast toned down in an attempt to make them harder to discriminate from each other. Additionally, the samples were presented in lists followed by lists of choice trials, again to reduce ceiling effects in the human version of the NHP task. Each stimulus subtended 10° of visual angle in width and 10° in height to the participant sitting facing the screen. The sample stimulus was always presented on the right top of the screen, positioned +12° horizontal and −12° vertical from the centre of the screen. The test stimuli were presented on the right bottom of the screen: One was positioned 0° horizontal and +5° vertical from the centre of the screen; and the other one was positioned +23° horizontal and +5° vertical from the centre of the screen. The background colour to the screen was white.

Participants sat with their eyes a distance of 25 cm from the screen, wearing earplugs in order to avoid audio disturbance of TMS pulses, resting their chins on a chin rest and their foreheads on a head holder to stabilize their head position throughout the experiment. They were instructed to respond to items by touching them on the screen and gestured their confidence ratings using their right hands. For example, they indicate by raising fingers (1, 2 or 3) whether their opinion corresponds to their being somewhat confident (1), moderately confident (2) or absolutely confident (3) in their judgement as to whether they considered the test stimulus to be old (i.e., presented before in the preceding list as a sample) or new (not seen before in the preceding list as sample). None of the samples in this task were used in more than one list so all stimuli were trial unique and hence comparable with the novel/trial‐unique stimuli in the NHP task.

rTMS was carried out using a biphasic Magstim Super Rapid^2^ magnetic stimulator (Magstim, Dyfed, UK) with a double 70‐mm figure‐of‐eight coil. The TMS coil was clamped in line with participants' assigned brain sites and localized at a 45° angle off the midline with the handle pointing to the posterior direction throughout the experiment.

#### 2.2.5 Behavioural task

**FIGURE 2:**
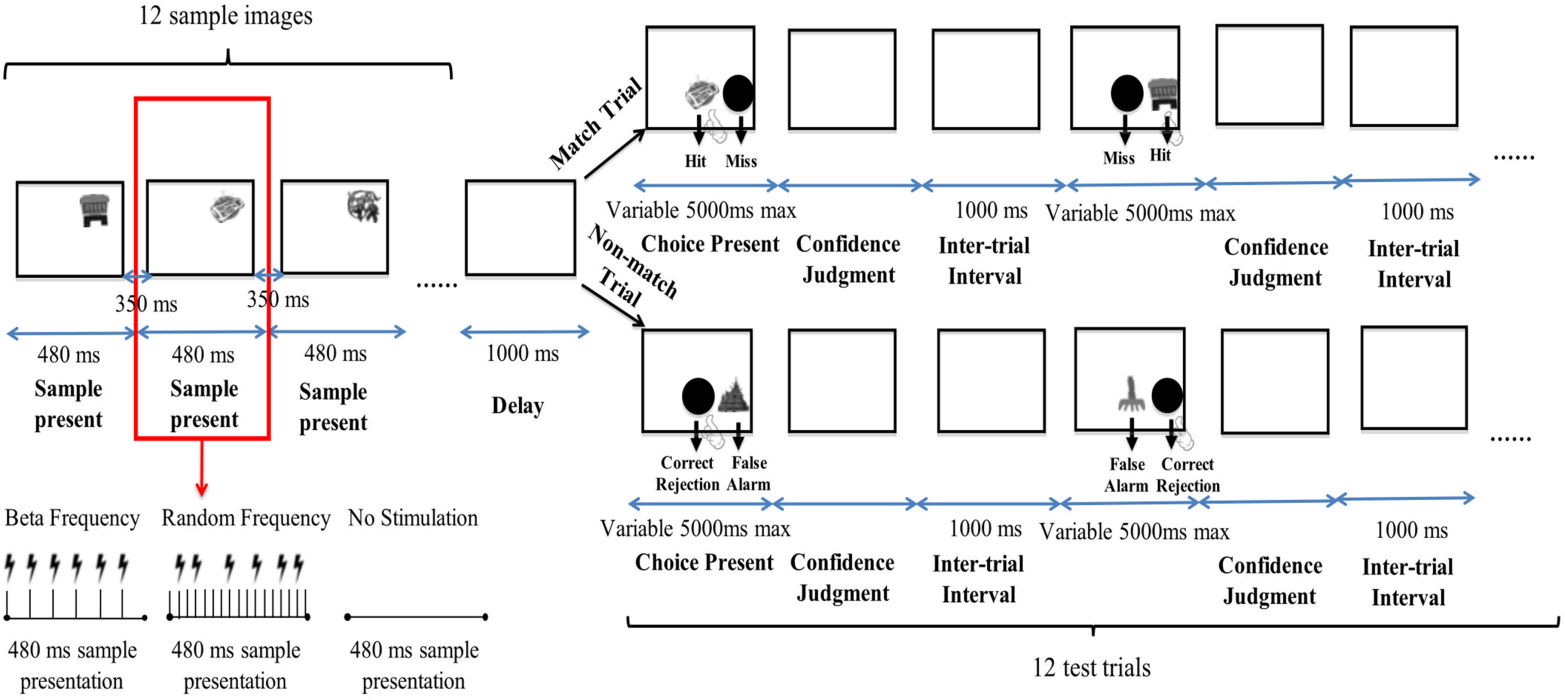
Human recognition memory task structure. The figure depicts the task structure for one block of trials in the transcranial magnetic stimulation (TMS) study. Twelve sample object images were sequentially presented for 480 ms with an interstimuli interval of 350 ms. Six repetitive TMS (rTMS) pulses (at beta frequency or at random frequency) or no stimulation were given during the sample presentation for four out of the 12 samples in the list (the different stimulation protocols were never intermixed within the same block of trials). Then, after a delay of 1,000 ms, 12 test trials followed (the right half of the figure shows the procedure for one of these 12). Just as in the NHP task in Figure 1a for Experiment 1, in ‘match trials’, the black circle was presented with an identical image to one of the preceding sample; in ‘nonmatch trials’, the black circle was presented with a novel stimulus not seen before. Participants were previously instructed to touch the test item if they remembered the test item was a match but to touch the black circle if they thought the test item was a nonmatch; accordingly, the responses could be separated into hits, misses, correct rejections and false alarms as indicted. Each test trial in this human version of the task lasted maximally 5,000 ms and ended with a confidence judgement indicated by the participant by their making a finger gesture as described in the main text. The intertrial interval was 1,000 ms

Prior to the experiment, participants were given instructions on how to perform the task and how to indicate their confidence judgements (see above), and then they were introduced to the behavioural task in a short training/practice session. This session composed of one list of 12 consecutive samples followed by its associated 12 consecutive choice/test trials albeit without any rTMS stimulation so to provide an opportunity to become familiarized with task procedure. We used lists in the human version (to avoid ceiling effects as in our pilot investigations). The experimental session itself contained three subsessions, with each subsession containing 15 blocks of trials. Participants took a 10‐ to 15‐min break between each subsession. Each block contained an encoding phase (i.e., a list of 12 sample images), and then a short delay (i.e., 1 s), and then a test phase (i.e., a list of 12 choice/test trials). The task structure is depicted, for one block, in Figure 2. During the encoding phase, 12 sample images were presented sequentially on the screen (individually for 480 ms each), with an interstimulus interval of 350 ms. Participants were instructed to try to remember each sample stimulus. After the sample phase (all 12 sample items) was completed, a blank screen was presented for 1 s (delay), and then the test phase commenced (all 12 test trials). Test trials were either ‘match trials’ or ‘nonmatch trials’. In each test trial, either an identical stimulus to one of the preceding 12 samples (i.e., ‘match trials’) was shown as a test stimulus, or a novel and previously unseen stimulus (i.e., ‘nonmatch trials’) was shown as a test stimulus, and that test stimulus appeared on the screen together with a black circle. The left/right position of the black circle and the test‐trial image were randomized between trials. The match and nonmatch trials were also counter balanced (6 of each per set of 12 choice/test trials) and were put in a random order. Just as in the NHP version of the task (Experiment 1), participants should touch/select the test stimulus if they thought it matched one of the 12 sample stimuli, or touch/select the standard ‘nonmatch button’ (i.e., the black circle) if they thought the test stimulus was not a match. Participants could touch the screen to make responses once the test image and black circle were on the screen, with a maximum responding duration set to 5,000 ms (i.e., test trial ended if participants did not make responses within 5,000 ms). After responding to the test image, participants were instructed to rate their confidence as to whether the test stimulus was new or old using a scale of 1–3 by making three different movements with their fingers, corresponding to somewhat, moderate and absolute confidence. Participants were further instructed to try to use the entire range of confidence responses as best they could and not simply select the extremes of confidence as defaults. The intertrial interval was 1,000 ms.

#### 2.2.6 rTMS protocol

Our LFP investigations in Experiment 1 revealed heightened alpha/low‐beta power in dlPFC when macaques viewed and encoded sample images in a similar recognition memory task; moreover, the extent of low‐beta power modulation was associated with success or error in subsequent macaque recognition memory performance. Thus, our rTMS stimulation in this human study in Experiment 2 was targeted to the encoding phase of the memory task. Our aim was to assess whether encoding phase‐related activity in that region at that time may be causally relevant for successful task performance in humans, despite species differences and some task differences as described.

In order to fall significantly below with the safety threshold guidelines for TMS (Rossi et al., 2009) to avoid seizure risks caused by too much sequential high‐frequency stimulation, a restriction was implemented that there were at least two nonstimulated stimuli in between every stimulated stimulus in each sample list of 12 items. Therefore, we determined all the possible combinations wherein fourout of 12 sample stimuli might be targeted, with at least two nontargeted intervening samples, and each block's sample phase took one of those schedules selected randomly each time. During beta‐stimulation blocks, each of the four rTMS targeted samples in the list (each with its duration of 480 ms as detailed above) was targeted with the delivery of six pulses every 80 ms (i.e., at 12.5 Hz). In the random‐stimulation blocks, the same total number of pulses (six) were delivered less regularly over the same period of presentation time (specifically, the 480‐ms sample presentation epoch was divided into 30‐ms intervals, and six of those, with the constraint that no two consecutive 30‐ms intervals could be chosen, were chosen at random to trigger one of the six TMS pulse deliveries). Each subsession contained 15 blocks, with each of the three ‘types’ of stimulation (i.e., beta‐stimulation, random‐stimulation and no‐stimulation) occurring five times per subsession, with the blocks randomly ordered. Each participant received the same sets of stimuli and same stimulation order. As there were three subsessions in total, each stimulation condition was repeated 15 times per participant, and so participants performed a total of 540 trials (i.e., three subsessions × 15 blocks per subsession × 12 trials per block).

#### 2.2.7 Data analysis

Behavioural effects of rTMS were evaluated based on DPSD theory model. According to this model, both familiarity and recollection contribute to recognition memory (Yonelinas, 2001, 2002). A recollection index (*R*) and familiarity index (*F*) were extracted by fitting the model to data using a standard approach that minimized the squared difference between the observed data and model predication in each cumulative confidence rating bin. Specifically, participants were asked to express their confidence in each of their decision using a 6‐point scale, where 1 implies *absolutely certain old*, 2 for *moderately certain old*, 3 for *slightly certain old*, 4 for *slightly certain new*, 5 for *moderately certain new* and 6 for *absolutely certain new*. The cumulative hit rate was then plotted against the cumulative false alarm rate to create the ROC plot of each participant. To be more specific for those not yet familiar with ROC plots in this context, the first point at the left‐hand side of the plot was the hit rate and false alarm rate at confidence level 6. After that, the second point was for the combination of confidence levels 6 and 5, the third point for confidence levels 6–4 and so on. The cumulative hit rate and false alarm rate of all confidence levels were constrained to 1.0. Accordingly, we had five coordinate points for each ROC plot.

The ROC data were analysed using the DPSD model. This model assumes recognition memory to be contributed towards by two distinct processes referred to as recollection and familiarity. For target‐ and lure‐stimulus trails with confidence levels equal to or larger than the *i*th confidence rating bin (i.e., *CL* ≥ *CL*_*i*_), the DPSD model predicts the cumulative false alarm rate in lure‐item trials and hit rate in target‐item trials as follows:

$$\begin{array}{c} p\left(\text{Lure}|\mathrm{CL}\geq {\mathrm{CL}}_{i}\right)=R_{n}+\left(1-R_{n}\right)*Ф\left(-c_{i}\right),\\ p\left(\text{Target}|\mathrm{CL}\geq {\mathrm{CL}}_{i}\right)=R_{0}+\left(1-R_{0}\right)*Ф\left(\frac{d{’}_{F}-c_{i}\;}{\sigma_{F}\;}\right). \end{array}$$

Briefly, in the target and lure distributions (Ф is the cumulative normal distribution), the parameters for the response criterion are designated as *c*_*i*_. In the target distribution, *R*_0_ is a target threshold parameter being labelled recollection of old stimuli, which is that the target items that have a strength above the *R*_0_ are classified as old. If the target item strength is below the *R*_0_, its classification is governed by the familiarity component of the model, which is a Gaussian distribution with the mean of *d'*_*F*_ and standard deviation of *σ*_*F*_.

In the lure distribution, *R*_*n*_ is a lure threshold parameter labelled ‘recollection’ of lures (Koen et al., 2017), while the classification for those with strength below the *R*_*n*_ threshold would be governed by a standard Gaussian distribution, with a mean of 0 and a unit standard deviation.

In our DPSD model, as we do not care about the lure distribution, we further make the *R*_*n*_ parameter is constrained to equal 0, and *σ*_*F*_ parameter is constrained to equal 1. Based on the above assumptions, the algorithm of DPSD model becomes:

$$p\left(\text{Lure}|\mathrm{CL}\geq {\mathrm{CL}}_{i}\right)=\;Ф\left(-c_{i}\right),$$

$$p\left(\text{Target}|\mathrm{CL}\geq {\mathrm{CL}}_{i}\right)=R_{0}+\left(1-R_{0}\right)*Ф\left(d{’}_{F}-c_{i}\right).$$

Each pair of the free parameters, *d'*_*F*_ (*F*) and *R*_0_ (*R*), defines a ROC curve. These parameters were obtained by minimizing the sum of squared residuals in both the abscissa and the ordinate of all the 5 points (that is, the sum of squared errors of prediction method for both axes). Based on the assumptions of the DPSD model, the range of *R* is between 0 and 1, while the range of *F* is between 0 and infinity (a theoretical maximum that is; practically, it is much less, around 3–4 as a maximum). If the best fit of the *R* value turns out to be negative in the initial fitting of ROC, we fixed it to be 0 (as a negative recollection score has no meaning beyond zero recollection) and refit the model to get *F*. In each stimulation type and in each stimulation site condition, a ROC is plotted cumulatively for each confidence level by the proportion of correct ‘old’ judgements against the proportion of incorrect ‘old’ judgements, and both *R* and *F* in each stimulation type and stimulation site condition were extracted from DPSD model.

#### 2.2.8 Statistical methods

All the statistical analyses on human behavioural data were carried out using SPSS software (IBM). The above calculated indices (i.e., *R* and *F*) were subjected to repeated measures analyses of variance (ANOVAs) with three levels of the within‐group factor stimulation type (beta‐stimulation, random‐stimulation and no‐stimulation) and with two levels of the between‐subject factor stimulation site (dlPFC, vertex). Similarly, accuracy in match trials (i.e., hit rate) was also assessed for each human participant in each stimulation condition and subjected to repeated measures ANOVAs with three levels of the within‐subject factor stimulation type (beta‐stimulation, random‐stimulation and no‐stimulation) and with two levels of the between‐subject factor stimulation site (dlPFC, vertex). Data were presented in raincloud plots to clearly illustrate each data point and distributions (Allen et al., 2019).

## 3 RESULTS

### 3.1 Experiment 1: NHP electrophysiological study

**FIGURE 3:**
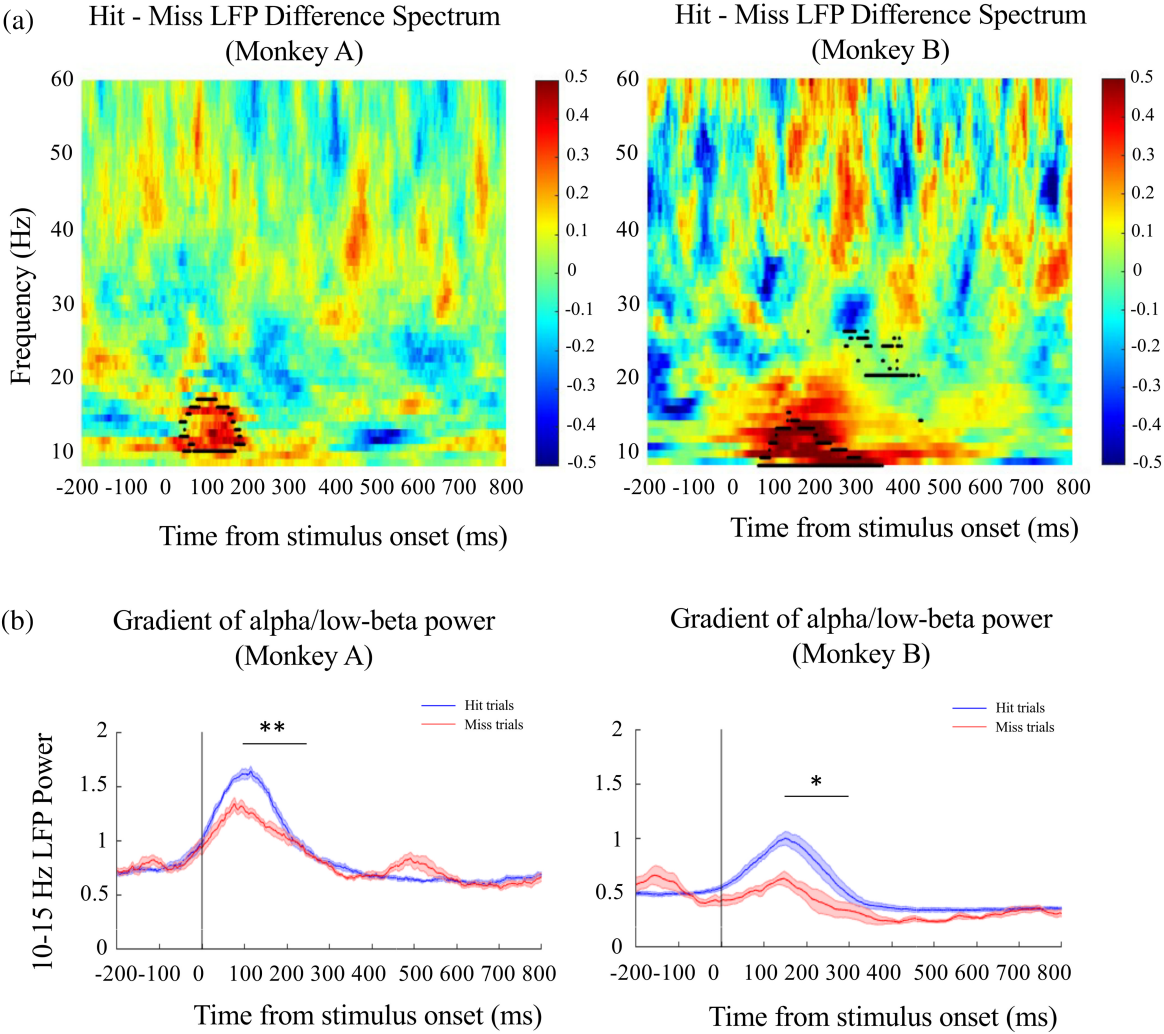
Time‐frequency spectrogram for induced local field potential (LFP) power and averaged LFP power in alpha/low‐beta band (10–15 Hz) from dorsolateral prefrontal cortex (dlPFC) in the sample presentation epoch in two nonhuman primates (NHPs) in Experiment 1. Panel (a): The areas of statistically significant differences of LFPs between pre‐ and post‐sample periods in macaque dlPFC were highlighted by a black outline in hit–miss contrast LFP difference spectrums; sample onset is indicated by 0 ms on this figure. Panel (b): Average time course of the LFP power in alpha/low beta (10–15 Hz) before and after sample onset (indicated by a vertical black line) in two NHPs. In each panel, blue line reflects averaged LFP power during sample presentation in hit trials; red line reflects averaged LFP power during sample presentation in miss trials. Error bars are the standard errors of the mean (SEMs) of each frequency band. The black line indicates the period when the gradient for each trial type was calculated. * and ** indicate a significant larger gradient for hit trial than miss trial at *p* < .05 and *p* < .01, separately

During the NHP recognition memory task, we focused only on the sample presentation/encoding phase. Our nonparametric permutation test described earlier (comparing post‐sample to pre‐sample phase) for Monkey A revealed a significant cluster ranging from 10 to 17 Hz increasing after the sample image onset in macaque dlPFC in sample presentations preceding hit choices in subsequent choice trials (two‐tailed permutation test, *p* < .05). The same nonparametric permutation test in Monkey B showed a significant cluster ranging from 8 to 15 Hz increasing after the sample onset in hit choice trials. In addition, there were some narrow frequency bands (14, 20, 22, 24–26 Hz) that significantly decreased (two‐tailed permutation test, *p* < .05). A separate nonparametric permutation test on miss choice trials revealed no significant change starting after the sample image onset in both monkeys (two‐tailed permutation test, *p* > .05). In hit choice trials, this significant increased post‐sample LFP power occurred immediately after the sample onset, which lasted circ. 100 ms in Monkey A and circ. 250 ms in Monkey B. Accordingly, the common frequency bands across two NHPs was 10–15 Hz. To better illustrate the difference between hit and miss trials in both monkeys, averaged LFP power in this common frequency band, we plotted the 10‐ to 15‐Hz hit versus miss spectra separately (Figure 3b) wherein it is clear to see that immediately after sample onset, a larger increase of alpha/low‐beta power (i.e., in this, 10‐ to 15‐Hz range plotted) was detected in hit trials than in miss trials in both monkeys (Figure 3b). A second consistent performance‐related finding between the two monkeys was identified: After the aforementioned transient BBA peak, the negative gradient of return to baseline (Figure 3b) was most pronounced in this frequency band (as assessed by plotting other bands, data not shown) and also markedly steeper in hit trials than in miss trials. This negative gradient after the peak in hit compared with miss trials was calculated across 150 ms for Monkey A (100–250 ms for Monkey A as its BBA peak at that frequency was around 100 ms of post‐sample). The negative gradient for Monkey B was calculated across 150 ms (150–300 ms for Monkey B as its BBA peak at this frequency was around 150 ms of post‐sample). Within the 10‐ to 15‐Hz band, we observed a more negative gradient in hit trials (mean gradients across 10–15 Hz were −5.70 × 10^−3^ in Monkey A and −3.75 × 10^−3^ in Monkey B) than in miss trials (mean gradients were −2.55 × 10^−3^ in Monkey A and −2.03 × 10^−3^ in Monkey B). The paired sample *t* test to compare the gradients in two trial types confirmed a more negative gradient in hit trials than in miss trials in both monkeys (Monkey A: *t*_[5]_ = −9.299, *p* = .0003; Monkey B: *t*_[5]_ = −3.922, *p* = .011). Our observations of these performance related differences in the 10‐ to 15‐Hz range in both monkeys led us to select an rTMS stimulation frequency that was exactly intermediate within this range (i.e., we chose low‐beta stimulation at 12.5 Hz). Importantly, this frequency of rTMS was well within the safety guidelines (with respect to duration and frequency considerations for repetitive stimulation) for humans (Rossi et al., 2009) in the manner in which we intended to use it in Experiment 2 in our homologous recognition memory task in humans.

### 3.2 Experiment 2: Human rTMS recognition memory study overview

Participants were highly accurate in their recognition memory task; mean performance and response time are shown in Table 1. We carried out a within‐block analysis and a between‐block analysis for hit rate (i.e., correct response rate in match trials), recollection and familiarity, separately. First, we carried out the within‐block analysis, specifically aiming to investigate the possible extent to which only match trials whose samples were targeted by rTMS might be affected by either beta stimulation or random stimulation. Alternatively, the effect of rTMS is more general than that and ‘spread’ its effect in a block, so affecting performance even on match trials whose samples were not targeted by rTMS given this task involved memories for lists of samples. Note that the no‐stimulation condition/blocks could not be included in this within‐block analysis as there was no differential targeting/no targeting of samples in the sample lists in these blocks. Hence, in the within‐block analyses, the aforementioned behavioural measurements were compared between the trials containing stimulated samples (*N* = 36 in beta‐stimulation blocks; *N* = 33 in random‐stimulation blocks) and trials not containing stimulated samples (*N* = 54 in beta‐stimulation blocks; *N* = 57 in random‐stimulation blocks). Next, the between‐block analysis aimed to investigate how the different stimulation types affected behavioural performance generally. In this analysis, the three aforementioned behavioural measurements were compared between the three stimulation conditions/blocks. For each participant, we included the following: all nonmatch trials (*N* = 90) in the three blocks; match trials whose samples were stimulated in beta‐stimulation blocks (*N* = 36) and in random‐stimulation blocks (*N* = 33); and all match trials in the no‐stimulation blocks (*N* = 90).

**TABLE 1:** Descriptive statistics: accuracy and response time of correct responses for each participant group across each stimulation condition

Brain area	Stimulation	Accuracy	Response time (ms)
dlPFC (*n* = 9)	Beta frequency	0.85 ± 0.02	1727 ± 147
	Random frequency	0.84 ± 0.01	1742 ± 157
	No stimulation	0.84 ± 0.02	1738 ± 154
Vertex (*n* = 9)	Beta frequency	0.88 ± 0.02	1,546 ± 66
	Random frequency	0.89 ± 0.02	1,503 ± 72
	No stimulation	0.88 ± 0.02	1,538 ± 75

Abbreviation: dlPFC, dorsolateral prefrontal cortex.

### 3.3 Within‐block analysis for the impact of rTMS on recollection and familiarity

To investigate the potential contribution of recollection and familiarity to the performance of the recognition memory task in humans, the recollection index (*R*) and familiarity index (*F*) were calculated for each participant in each of the three stimulation types in the standard way described earlier for these tasks. We first conducted our more contextually sensitive ‘within‐block’ analyses wherein we calculated within‐block (and hence more controlled for time and order effects) comparisons of *R* and *F* indices for trials that used samples that had occurred in the sample phase that was targeted with rTMS (denoted +) versus trials that used samples that occurred in the sample phase that were not targeted with rTMS (denoted −); hence, we refer to these four indices as *R*+, *R*−, *F*+ and *F*−. These indices cannot be calculated for the no‐stimulation block as all trials in that block are without rTMS so the within‐block analyses are restricted to beta‐stimulation and random‐stimulation blocks. Specifically, we investigated whether the within‐block numerical difference between *R*+ and *R*−, and also between *F*+ and *F*−, differed between blocks, that is, whether these differences themselves depended upon whether samples were targeted with beta versus random stimulation. The aim was to determine whether any dlPFC beta‐stimulation effect on *R* or *F* might, in these sensitive within‐block analyses, be statistically significantly different from the random‐stimulation effects on these indices, so to address whether the rTMS effect may be beta frequency specific to any degree (i.e., to the limited extent that we can do in this study with only two stimulation protocols that is).

Accordingly, a repeated measures ANOVA, with rTMS targeted (two levels: *R*+ and *R*−) and stimulation type (two levels: beta‐stimulation block and random‐stimulation block) as within‐subjects factors, and with stimulation site as the between‐subjects factor (two levels: dlPFC and vertex), was conducted on recollection indices. A significant two‐way interaction between rTMS targeted and stimulation type (*F*_[1,16]_ = 4.659, *p* = .046, *η* ^2^ = .226) and another significant interaction between rTMS targeted and stimulation site (*F*_[1,16]_ = 8.535, *p* = .010, *η* ^2^ = .348) were detected, prompting further analysis. We then carried a repeated measures ANOVA on recollection indices observed in dlPFC, with rTMS targeted (two levels: *R*+ and *R*−) and stimulation type (two levels: beta‐stimulation block and random‐stimulation block) as within‐subjects factors. It showed a significant main effect of rTMS targeted (*F*_[1,8]_ = 7.721, *p* = .024, *η* ^2^ = .491) and a significant interaction between stimulation type and rTMS targeted (*F*_[1,16]_ = 6.508, *p* = .034, *η* ^2^ = .449), which prompts follow‐up analysis. A paired *t* test between beta‐ and random‐ stimulation for *R*+/*R*− indicated a significant decrease of *R*+ compared with *R*− in beta‐stimulation (*t*_[1,8]_ = −5.502, *p* = .001), but this was not true for random‐stimulation (*t*_[1,8]_ = 0.119, *p* = .908). Then, with respect to vertex, the repeated measures ANOVA on the recollection indices with rTMS targeted (two levels: *R*+ and *R*−) and stimulation type (two levels: beta‐stimulation block and random‐stimulation block) as within‐subjects factor did not show a main effect of rTMS targeted (*F*_[1,8]_ = 3.176, *p* = .113, *η* ^2^ = .284) nor an interaction effect (*F*_[1,8]_ = 0.996, *p* = .347, *η* ^2^ = .111).

The corresponding *F*+/*F*− analysis showed a three‐way interaction among rTMS targeted, stimulation site and stimulation type (*F*_[1,16]_ = 5.604, *p* = .031, *η* ^2^ = .259), which prompts further analysis. A repeated measures ANOVA on familiarity indices observed in dlPFC, with rTMS targeted (two levels: *F*+ and *F*−) and stimulation type (two levels: beta‐stimulation block and random‐stimulation block) showed neither the main effect of rTMS targeted (*F*_[1,8]_ = 0.012, *p* = .917, *η* ^2^ = .001) nor an interaction effect (*F*_[1,8]_ = 3.060, *p* = .118, *η* ^2^ = .277) was significant (see Figure 5c,d). In vertex, neither a significant main effect of rTMS targeted (*F*_[1,8]_ = 4.618, *p* = .064, *η* ^2^ = .366) nor the two‐way interaction (*F*_[1,8]_ = 2.551, *p* = .149, *η* ^2^ = .242) was observed for the *F*+/*F*− statistic.

**FIGURE 4:**
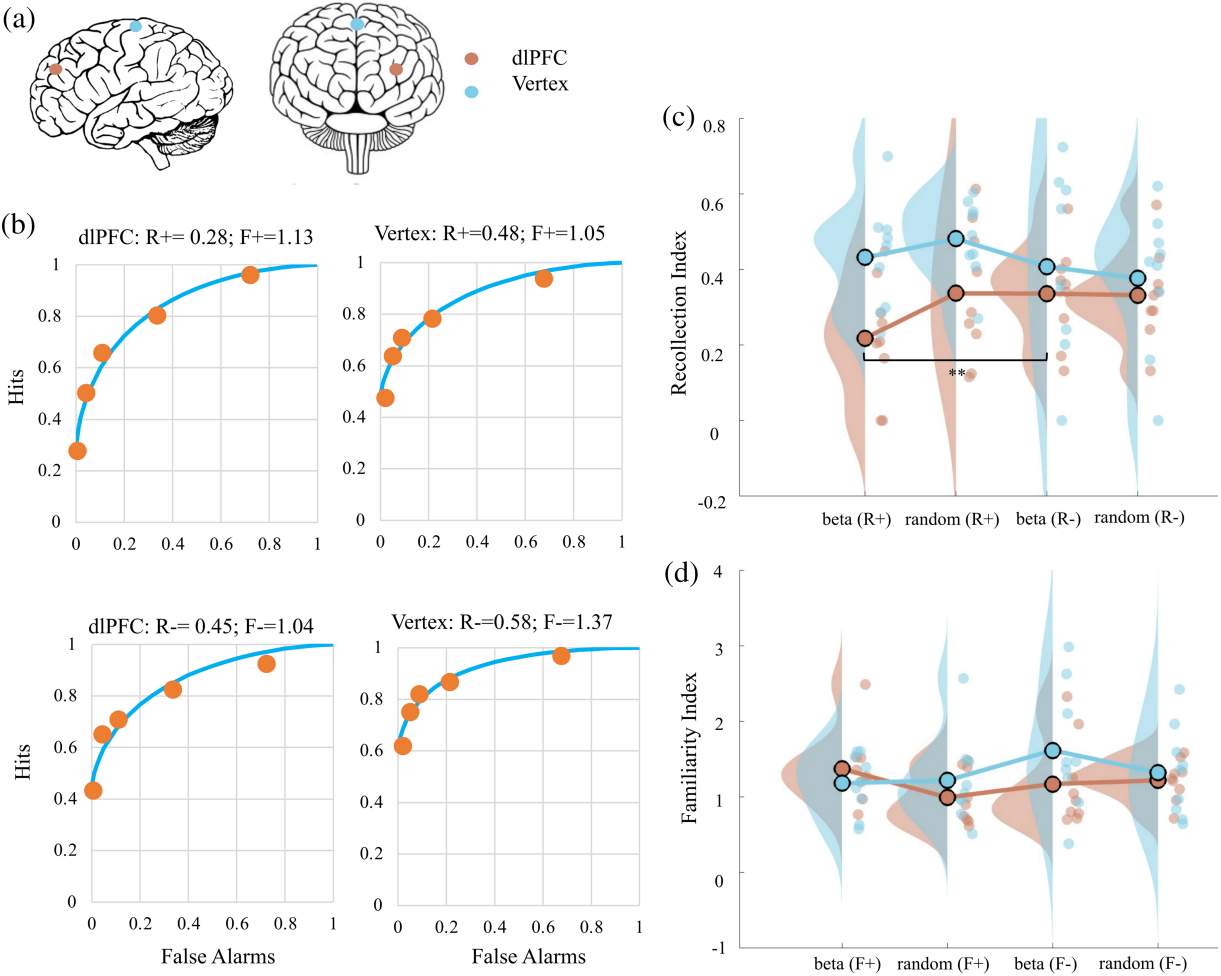
Stimulation sites for each group of human participants and within‐block analysis for the effects of transcranial magnetic stimulation (TMS) upon recollection and familiarity in each human participant group in Experiment 2. Panel (a): Lateral and frontal view of the human brain depicting stimulation sites for repetitive TMS (rTMS) to dorsolateral prefrontal cortex (dlPFC) (red) and vertex (blue). Panel (b): Averaged receiver‐operating characteristics (ROCs) fitted for the recognition data under beta frequency stimulation with samples targeted with rTMS (+) or without rTMS (−). Accordingly, the *R* and *F* indices were labelled as *R*+, *R*−, *F*+ and *F*−. The ROCs depict decreased recollection under beta frequency stimulation with rTMS to dlPFC (but no change for vertex). Panels (c) and (d): Raincloud plots presenting the recollection index (Panel c) and familiarity index (Panel d) in dlPFC (red) and vertex (blue) in *R*+, *R*−, *F*+ and *F*− conditions. Circles are individual data; circles with black edge colour show the average of group data; distributions show probability density function of data points. ** indicates *p* < .01

### 3.4 Between‐block analysis for the impact of rTMS on recollection and familiarity indices

We then carried out a between‐block analysis on *R* and *F* in the three stimulation conditions/blocks to investigate how the different stimulation types affected behavioural performance generally and accordingly with the inclusion of the no‐stimulation block control on this purpose. A mixed‐model repeated measures ANOVA was conducted on the indices for the *R* and *F* indices separately, each with stimulation site (two levels: dlPFC and vertex) as the between‐subjects factor and stimulation type (three levels: beta‐stimulation blocks, random‐stimulation blocks and no‐stimulation blocks) as the within‐subject factor.

For the *R* index, we found a significant main effect of stimulation type (*F*_[2,32]_ = 3.756, *p* = .034, *η* ^2^ = .190), main effect of stimulation site (*F*_[1,16]_ = 8.330, *p* = .011, *η* ^2^ = .342) and a significant linear trend evidenced by a significant within‐subject linear contrast component of the interaction (*F*_[1,16]_ = 4.490, *p* = .050, *η* ^2^ = .219). The latter prompted further scrutiny by a repeated measures ANOVA to each stimulation site independently. Therefore, we next applied repeated measures ANOVAs to dlPFC site with the aforementioned stimulation type as the within‐subjects factor. Although the main effect of stimulation type was only marginally significant (*F*_[2,16]_ = 4.432, *p* = .055, *η* ^2^ = .356; with Greenhouse–Geisser correction due to a significant Mauchly's test of sphericity), there was a highly significant effect of the linear trend component of stimulation type (*F*_[1,8]_ = 21.547, *p* = .002, *η* ^2^ = .729), which prompted further scrutiny by post hoc pairwise *t* tests with Bonferroni correction. These revealed a significant suppression of the *R* index under beta‐stimulation compared with *R* under no‐stimulation (*p* = .005), although no significant difference of the *R* index between beta‐stimulation and random‐stimulation was found (*p* = .272). Parallel analyses conducted for the vertex site showed that neither the main effect (*F*_[2,16]_ = 0.691, *p* = .516, *η* ^2^ = .079) nor the linear trend of stimulation type (*F*_[1,8]_ = 0.046, *p* = .836, *η* ^2^ = .006) was significant, so no follow‐up analyses were required.

For the *F* index, the parallel repeated measures ANOVA with stimulation type as the within‐subjects factor and stimulation site as the between‐subjects factor showed that neither the main effects (stimulation type: *F*_[2,32]_ = 0.966, *p* = .391, *η* ^2^ = .057; stimulation site: *F*_[1,16]_ = 0.170, *p* = .686, *η* ^2^ = .011) nor their interaction (*F*_[2,32]_ = 1.670, *p* = .204, *η* ^2^ = .095) was significant, so no follow‐up analyses were required.

**FIGURE 5:**
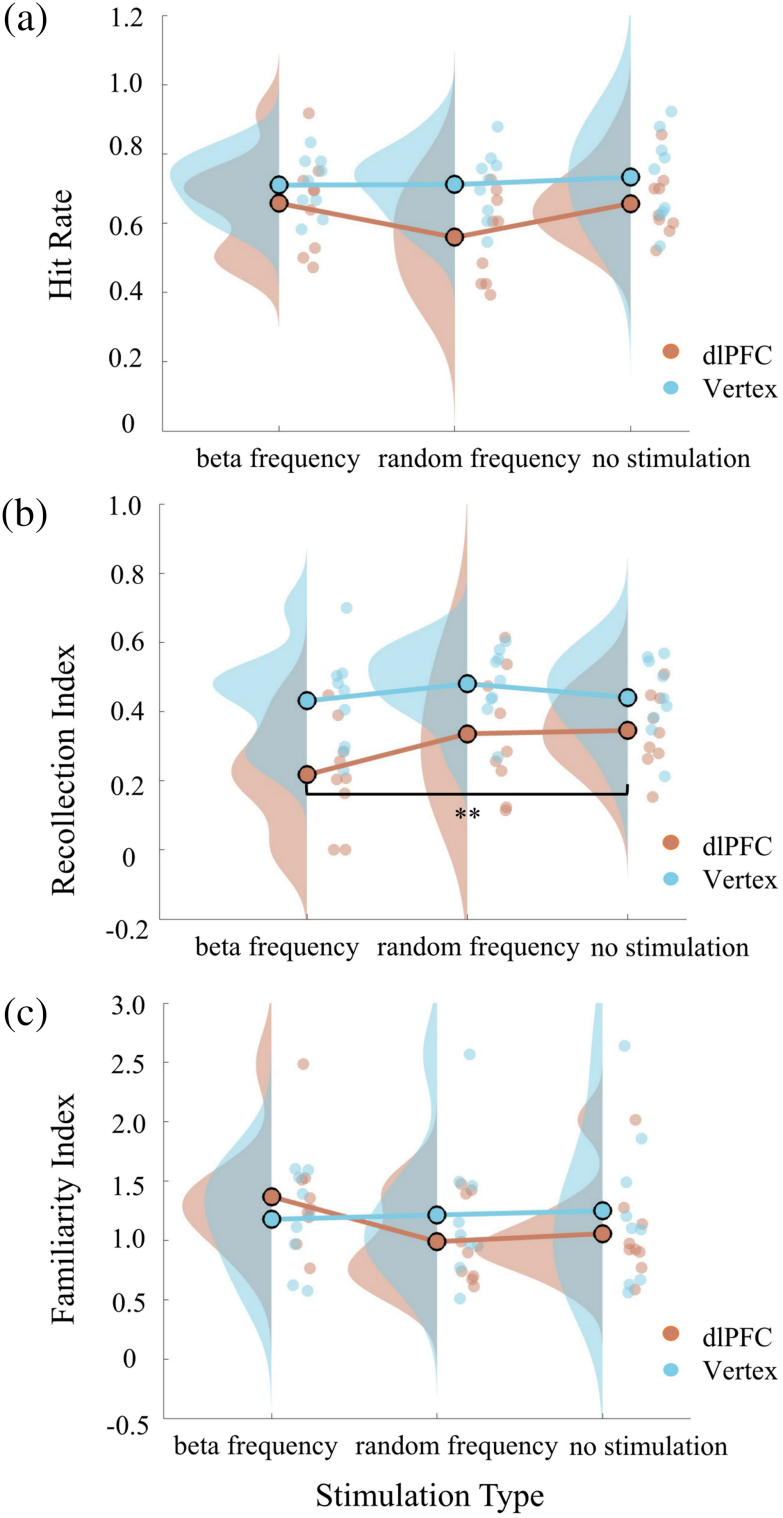
Between‐block analysis for the effects of transcranial magnetic stimulation (TMS) upon hit rate, recollection and familiarity indices in Experiment 2. Panels (a)–(c): Raincloud plots presenting the hit rate in match choice trials (Panel a), the mean recollection index (Panel b) and familiarity index (Panel c) in dlPFC (red) and vertex (blue) under beta frequency stimulation, random frequency stimulation and no stimulation. Circles are individual data; circles with black edge colour show the average of group data; distributions show probability density function of data points. ** indicates *p* < .01

In summary, these between‐block analyses indicate (see Figure 5b,c) that the *R* index was significantly suppressed by dlPFC beta rTMS but the *F* index in contrast was not. However, the aforementioned within‐block analyses (Figure 4c,d) lend support to the notion that the beta‐stimulation on the *R* index in dlPFC does not in fact generalize to all rTMS stimulation parameters (i.e., in our case to the random‐stimulation condition). We note the numerical trend (albeit nonsignificant) seen in the between‐block analyses (see Figure 5b), which at least raises the possibility of a beta frequency effect (given the between‐block analysis is less sensitive to context as described above). Naturally, we must remain cautious on this interpretation; the sample size per target area while limited was in fact similar to that used in a previous study that showed significant group difference in rTMS studies (Boschin et al., 2017) and was deemed a priori to have sufficient power to reveal large differences between groups (as verified in this study and in that previous rTMS study) as well as within practical limits imposed by the context of the student research projects we supervised for this study.

### 3.5 Impact of rTMS on hit rate

Finally, for our analyses of hit rate, we focused only on performance in match trials because in our task design, our nonmatch trials contain no old stimuli and so cannot facilitate hit responses. We similarly carried out within‐block analyses to address the question of whether our rTMS protocols might be having effects specific to choice trials that contained those samples (only four out of 12 samples in the each sample list were targeted by rTMS) that were specifically targeted by rTMS or whether rTMS to those four out of 12 samples might be having a broader spread of effect upon performance across the block in general. We calculated hit rate for match trials that contained samples that had been targeted by rTMS in the sample list phase (denoted +) and compared that with the hit rate for match trials that did not contain samples targeted by rTMS in the sample list phase (denoted −). The hit rate data were subjected to an ANOVA with the within‐subjects factor ‘targeted’ (two levels: hit rate + versus hit rate −), the within‐subjects factor stimulation type (two levels: beta‐ and random‐stimulation blocks) and stimulation site as the between‐subjects factor (two levels: dlPFC and vertex). We found that there was a significant main effect of stimulation type on this hit rate measure (*F*_[1,16]_ = 5.388, *p* = .034, *η* ^2^ = .252) and also a significant three‐way interaction between stimulation type and targeted and stimulation site (*F*_[1,16]_ = 6.849, *p* = .019, *η* ^2^ = .300) which prompted follow‐up investigation of each stimulation site separately, which revealed that the main effect of the factor ‘target’ was not significant in any of the two regions (dlPFC: *F*_[1,8]_ = 1.156, *p* = .314, *η* ^2^ = .126; vertex: *F*_[1,8]_ = 0.277, *p* = .613, *η* ^2^ = .034) nor were interactions with stimulus type (dlPFC: *F*_[1,8]_ = 2.767, *p* = .135, *η* ^2^ = .257; vertex: *F*_[1,8)_ = 4.679, *p* = .062, *η* ^2^ = .369).

We also carried out between‐block analyses using a mixed‐model repeated measures ANOVA, with stimulation site as the between‐subjects factor (two levels: dlPFC and vertex) and stimulation type as the within‐subject factor (three levels: beta‐stimulation blocks, random‐stimulation blocks and no‐stimulation blocks) conducted on hit rate. The analysis showed that neither the main effects (i.e., stimulation site and stimulation type) nor their interaction was significant (main effect of stimulation site: *F*_[1,16]_ = 4.162, *p* = .058, *η* ^2^ = .206; main effect of stimulation type: *F*_[2,32]_ = 3.015, *p* = .063, *η* ^2^ = .159; interaction: *F*_[2,32]_ = 2.069, *p* = .143, *η* ^2^ = .114) with respect to hit rate (Figure 5a). In summary, neither the within‐block nor the between‐block analyses of hit rate indicated that rTMS stimulation had any effects on accuracy in match choice trials, be they those containing stimulated samples and/or those containing no‐stimulated samples, in either beta‐ or random‐stimulation conditions.

## 4 DISCUSSION

### 4.1 Attentional‐synchronization and mnemonic‐desynchronization hypotheses pertaining to BBA

In Experiment 1, we conducted multielectrode direct extracellular recordings in two NHPs to determine what spatial and temporal parameters of LFP power existed in macaque dlPFC during the sample stimulus presentation phase in an object recognition memory/working‐memory task previously shown to be amenable to comparative human and NHP behavioural investigation (Wu et al., 2020). Then, in Experiment 2, to assess causality, we employed a human version of this paradigm and spatial and temporal rTMS parameters derived from LFP observations in monkeys from Experiment 1. The electrophysiological observations in Experiment 1 revealed two performance related differences in the LFP signal. First, an early transient peak in alpha/low‐beta power in the 10‐ to 15‐Hz range during sample stimulus presentation was greater in trials wherein that sample subsequently ended up in correct ‘hit’ trials than in trials wherein the sample ended up in incorrect ‘miss’ trials (Figure 3a). Second, after the aforementioned 10‐ to 15‐Hz peak in LFP power, its subsequent rate of decline was more rapid in in trials wherein that sample ended up in hit trials compared with those ending up in miss trials (Figure 3b). These transient BBA power observations led us to propose distinct hypotheses in line with some aspects of the existing BBA literature. First, in line with attentional‐synchronization hypotheses, we hypothesized that 12.5‐Hz rTMS (we chose 12.5 Hz as our stimulation frequency as it was intermediate in the significant 10‐ to 15‐Hz range common to both NHPs) during sample presentation in humans might artificially entrain BBA and enhance attention resulting in memory improvement for those targeted samples later at test. Second, in line with mnemonic‐desynchronization hypotheses, we alternatively hypothesized that targeting human dlPFC with12.5‐Hz rTMS during sample presentation may impair subsequent memory for those targeted sample via purported rTMS beta entrainment interfering with normal desynchronization processes that support normal memory.

Turning to the first hypothesis, the transient increased low BBA power around the time of sample stimulus presentation, lasting at least 100–250 ms and had an early onset and in hit trials appeared to start to rise at or possibly even slightly before sample onset (Figure ). Previous electrophysiological investigations in NHPs have also found early involvement of dlPFC in the representation of salient stimuli. For example, population visual responses with latencies as early as 42 ms have been linked to bottom‐up visual attention (Buschman & Miller, 2007; Katsuki & Constantinidis, 2012), and signals with ~120‐ms latencies have been linked with top‐down visual attention (Buschman & Miller, 2007). In humans too, MEG studies have shown 8‐ to 14‐Hz activity in frontal regions for perceived stimuli lasting 30–150 ms after stimulus onset (Palva et al., 2005; Palva & Palva, 2011). These aforementioned latencies/durations are all well within the time frame of the heightened low BBA power observed in Experiment 1. Therefore, although our early signal may be related to mnemonic encoding per se (which we'll consider later), we cannot rule out that it relates to early visual and/or attentional processes that secondarily benefited sample stimulus encoding and subsequent memory performance. Accordingly, our first hypothesis for Experiment 2 was that 12.5‐Hz rTMS to dlPFC throughout sample stimulus encoding (in our study the sample epochs last 480 ms) might serve to entrain or strengthen these early attentional or perceptual processes. By similar logic, we reason that our randomly timed pulses of rTMS (same number of pulses and same total duration) would be disruptive to any such entrainment and would not have the same effect. Hence, we hypothesized beta, but not random, rTMS stimulation during sample presentations might enhance their subsequent memory at test. Accordingly, our control random stimulation by this hypothesis might be expected to either leave recognition intact or even disrupt subsequent memory for stimulated samples by preventing efficient attentional or perceptual processes. These hypotheses were clearly not supported. We found no beta rTMS stimulation effect on hit rate (Figure 5a), and random rTMS stimulation did not significantly impair performance either.

Turning to the second hypothesis, some human studies support the theory that desynchronization of alpha/beta power is necessary for optimum mnemonic encoding of stimuli. For example, a *decrease* of alpha/beta LFP power in frontal and parietal cortex correlated with *successful* memory encoding and retrieval (Hanslmayr et al., 2009, 2011; Khader & Rösler, 2011; Waldhauser et al., 2012). In accordance with this information by desynchronization theory (Hanslmayr et al., 2012), decreases in the alpha/beta frequency band are presumed to reflect a desynchronization of local neural assembles, and the rationale, based on information theory, is that synchronization of neural firings reduces the richness of information transfer that results in information redundancy. In contrast, desynchronization of neuronal firing leads to more potential for carrying information to improve the efficiency of neural communications (Hanslmayr et al., 2012). Indeed, it has also been shown previously using combined EEG‐rTMS that only stimulation at beta frequency produces an endogenous beta echo and impairs memory formation (Hanslmayr et al., 2014). In our NHPs in Experiment 1, we observed an association between rate of low BBA power reduction and memory performance wherein BBA declined more rapidly in hit trials than in miss trials (Figure 3b). Hence, our distinct second set of hypothesis for Experiment 2 was that our exogenously provided low‐beta (12.5 Hz) stimulation might similarly impede natural beta desynchronization processes associated with better memory performance at test. Our control random stimulation, according to this idea, would not be expected to entrain beta echo, would not be expected to interfere with desynchronization and hence would not be expected to affect subsequent memory for samples targeted with random rTMS. This hypothesis was supported only to a degree by Experiment 2. Our within‐block analysis found a suppression of *R*+ index by 12.5‐Hz rTMS stimulation in dlPFC compared with *R*− in beta stimulation, an effect not observed in random stimulation; and in our between‐block analyses, we observed significant effects of this low‐beta stimulation on subsequent recollection of samples. The effect that was on recollection was discerned from fitting standard ROC‐based and dual‐process theory models to the data (Figure 4b). We did not find any effect of the 12.5‐Hz rTMS stimulation on the familiarity indices when targeted to dlPFC (Figure 5c). As expected, our random frequency control stimulation was without effect on either recollection or familiarity indices either at dlPFC or at vertex (Figure 5b,c).

### 4.2 Potential mechanisms of BBA in humans and NHPs based on desynchronization theory

The purported mechanistic accounts of information by desynchronization theory remain an open question, but candidate mechanisms have been suggested. These include decreased low frequency power acting as a gating mechanism for increased spiking rates and/or reduction in the unreliability of neural code due to a reduction of ‘noise correlation’ induced by synchronized low frequency synchronized firing and/or decorrelated neural activity enhancing neuronal encoding capacity, for example, by allowing flexible phase adjustments associated with temporal encoding (Hanslmayr et al., 2016). If a robust bridge between NHP and human performance could be found, then future work in NHPs could evaluate these purported mechanisms more directly by recording simultaneously from multiple electrodes in multiple regions and combing that with targeted interventions (e.g., direct electrical stimulation through implanted electrodes as opposed to rTMS with relatively low spatial selectivity). However, Experiments 1 and 2 are more a proof of principle for such species bridging studies as clearly some significant limitations exist in interpretation as to whether the information by desynchronization theory really provides the most parsimonious account of our data as our LFP observations and stimulation parameters differ from those of Hanslmayr and colleagues' studies in a number of identifiable ways.

One key difference is that although our task epoch aligned macaque LFP, data look broadly like data associated with human LFP observed in desynchronization theory aligned studies (e.g., compare our Figure 3a right panel LFP spectrogram, with the spectrogram shown in Fig. 1a of Hanslmayr et al., 2012), there are also significant differences. Primarily, our BBA power peaked after the sample stimulus then quickly declined and returned to baseline (Figure 3a,b); in contrast, in Fig. 1a of Hanslmayr et al. (2012), their power decrease in BBA associated with the information by desynchronization hypothesis declines significantly *below* pre‐sample baseline. Whether this simply reflects species‐specific differences in baseline and endogenous rhythms pre‐sample remains to be investigated. There is some evidence that such differences may not be entirely unexpected as it is known that functionally relevant LFP power may be target area specific, task specific and/or stimulus specific to a degree (Burgess & Gruzelier, 2000).

A second key difference is that our rTMS stimulation parameters differed. This is inevitable because we explicitly set out to test causal effects of rTMS stimulation using parameters derived directly from our LFP observations in NHPs in Experiment 1 and the epochs inherent in our task structure. Hence, we used an rTMS protocol of six pulses at 12.5 Hz to last the 480‐ms sample stimulus presentation epoch, targeted to the homologous human brain region from which we recorded LFPs in macaque dlPFC. In contrast, Hanslmayr et al. (2014) used a beta‐stimulation protocol of 18 pulses at 18.7 Hz targeted to ventrolateral PFC and commencing 0.5 s after encoding commenced (compared with immediately upon sample presentation in our study). Again, our aim was not to investigate previously used rTMS parameters employed in distinctly different tasks; rather, it was to investigate new human rTMS parameters derived directly from our NHPs in our task that is known to reveal similar performance profiles in macaques and humans in the broad area of recollection/recollection‐like and familiarity‐based contributions to stimulus recognition memory (Wu et al., 2020). Hanslmayr et al. (2014) additionally showed that only rTMS at beta frequency produced beta echo: Their EEG data confirmed endogenous beta echo was driven after 18.7‐Hz rTMS entrainment but not after 6.8‐ or 10.7‐Hz stimulation. An obvious practical limitation of our Experiment 2 is we had no parallel online EEG recording available and therefore we could not validate that our rTMS protocol similarly entrained beta echo. Although the likelihood of beta entrainment/echo in our own study is promoted by consideration that rTMS only at beta frequency produces beta echo in Hanslmayr et al. (2014), their beta rTMS stimulation was at a higher frequency than ours (i.e., their 18.7 Hz vs. our 12.5 Hz), Moreover, their lower frequency stimulation (i.e., their 10.7 Hz, which is closer to our 12.5 Hz) failed to evidence echo. Hanslmayr et al. (2014) suggested that the selectivity of echo entrainment in their own paradigm to certain stimulation frequencies could be inherently related to the degree of similarity of the exogenously driven frequency with natural endogenous frequencies that are task relevant within specific target area. This brings us back to our preceding argument then, namely, that our rTMS parameters were derived from direct electrophysiological recordings of performance‐related BBA in NHPs, in homologous brain region, and on a similar task to that used our human study, for which performance profiles are similar in some respects. Therefore, if task‐associated LFP power is conserved across species to any degree (but see Buzsáki et al., 2013), then an exogenous BBA revealed in Experiment 1 in NHPs might indeed be associated with entrainment and echo in humans performing a similar task. This remains speculative and requires investigation in parallel EEG and TMS studies in humans alongside electrophysiological and stimulation studies in NHPs. We end this section by noting that the information by desynchronization hypothesis is not without support from NHP studies. Holmes et al. (2018) showed a dissociation between LFP beta power and mnemonic tuning and despite being in a spatial task is consistent with desynchronization aiding memory stability. Compte et al. (2003) showed that neural attractor network modelling in the context of delayed response task performance in NHPs also suggests mechanisms by which a drop in LFP power may relate to memory processing. Asynchronous networks can maintain memory coding for longer durations, whereas synchronization leads to overall instabilities incompatible with stable memory‐coding states (Compte et al., 2003). According to these models too, frontal memory networks desynchronize to preserve memory states.

### 4.3 Potential mechanisms of recollection and familiarity

We now move on to consider the extent to which our results pertain to recollection and familiarity per se. One consideration is whether ROC‐based analyses and associated extraction of recollection and familiarity indices based on DPSD models are appropriate for the short‐term memory task we used. This ROC approach has in fact long been adopted across wide‐ranging delay lengths in the literature, ranging from very short‐term recognition (i.e., several seconds to minutes) in humans and animals (Guderian et al., 2011; Wu et al., 2020), short‐term recognition (i.e., ~10–30 min) in humans and animals (e.g., Cipolotti et al., 2006; Fortin et al., 2004; Sauvage et al., 2008; Turriziani et al., 2010; Yonelinas, 2001) and intermediate to long‐term recognition memory (i.e., 1 h to several weeks) in humans (Wais et al., 2006). Our between‐block analyses in Experiment 2 showed that 12.5‐Hz rTMS stimulation to dlPFC significantly diminished R compared with no stimulation (Figure 5b), but a numerical trend failed to show that beta‐stimulation to dlPFC significantly diminished R compared with random‐stimulation. However, our more sensitive within‐block analyses suggest that the beta‐stimulation effect on the *R* index in dlPFC did not generalize to the random‐stimulation condition. Although further work is clearly required to determine the degree of frequency specific effects (both for 12.5 Hz and other frequencies), these initial investigations already show that rTMS stimulation to human dlPFC during sample stimulus viewing can produce effect upon recognition that affect ROCs in ways the literature interpret as recollective deficits. Moreover, these deficits exist in our very short‐term memory task. Previous studies investigating recollection/familiarity in PFC using rTMS have found, for example, that 20‐Hz rTMS targeted to a slightly more rostral region of left dlPFC (likely BA9) during stimulus encoding impaired retrieval of complex visual scene memory (Rossi et al., 2001), and impaired both recollection and familiarity of faces (Turriziani et al., 2008). Our Experiment 2, in contrast, shows that at least for visual objects their recollection and familiarity may be dissociated, while hit rate per se remains unaffected.

The above finding in Experiment 2 naturally provokes the question as to whether NHPs performing the similar task in Experiment 1 also draw upon recollective‐like and familiarity‐based processes to mediate performance in their short‐term recognition memory task. The current study cannot address this directly as we had no means to assess confidence in responses in our two NHPs needed in order to fit DPSD models to the data. But that was not our aim as other dedicated studies have already done this and succeeded in fitting DPSD models and adopted ROC approaches to derive recollection and familiarity indices in rodents (Fortin et al., 2004; Sauvage et al., 2008) and in NHPs (Guderian et al., 2011). Other studies have taken alternative approaches and dissociated fast familiarity processes from slow recollective processes in macaques similar to observations in humans (Basile & Hampton, 2013; Wu et al., 2020), and indeed, one recent study cross‐validated the ROC‐based derivation of recollection and familiarity indices with the FF/SR approach in humans (Wu et al., 2020). Importantly, for the current discussion, those studies were done in the context of the same short‐term recognition memory tasks used to investigate working memory in macaques and humans as used here in Experiments 1 and 2. On balance, we prefer to infer our macaques probably have recollective‐like processes contributing to their task in Experiment 1 just as humans do in their task in Experiment 2. There is of course a healthy and robust debate about these issues beyond the scope of this discussion to review (e.g., Allen & Fortin, 2013; Basile & Hampton, 2011; Eichenbaum et al., 2005, 2008; Wixted & Squire, 2008).

In thinking about recollection, arguably one of the most important concepts to consider with respect to BBA is its potential role in supporting neural communication. The ‘communication through coherence’ hypothesis proposes that synchronized neural oscillations within a particular brain region, or between distant brain areas, provides important mechanisms for mediating cognition via facilitating communication/interactions (Fries, 2005). This general concept is one of interaction between spikes and oscillatory LFPs, and brain areas fluctuating between frequencies and/or phase of oscillations with respect to each other, in order to ‘tune‐in’ (Fell & Axmacher, 2011; Fries, 2015). A combined fMRI‐EEG study indicates that low‐frequency oscillations support the PFC‐hippocampal network dynamics during recollection process (Herweg et al., 2016). Another human EEG study suggests the importance of interactions between low‐ and high‐frequency oscillations in memory retrieval (Köster et al., 2014). A ‘multieffect multinuclei’ model has also been proposed (Aggleton et al., 2011), whereby direct and indirect inputs to PFC from the hippocampus were associated with recollection process, while connections to PFC from perirhinal cortex and mediodorsal thalamic nucleus were related to familiarity process. Additionally, a three‐circuit model of neural oscillations between PFC and MTL via thalamus proposes PFC and hippocampus interact through theta oscillations (4–8 Hz) in support of core functions related to successful memory encoding and retrieval processes (Ketz et al., 2015). The human neuropsychology and neuroimaging literature also highlight the involvement of both the hippocampus (Bowles et al., 2010; Eichenbaum et al., 2007; Ranganath et al., 2004; Skinner & Fernandes, 2007; Yonelinas et al., 2002) and PFC (Wheeler & Stuss, 2003) in recollection. Hence, theta oscillations within a PFC‐hippocampus network may be essential for recollection process. It is known that theta rTMS over dlPFC decreases functional connectivity between dlPFC and hippocampus during working memory (Bilek et al., 2013). In this context, the external 12.5‐Hz rTMS over dlPFC in Experiment 2 may disrupt communications between PFC and hippocampus providing a plausible mechanism by which our rTMS effects decreased recollection. These extended circuit models of dlPFC and hippocampus interactions in recollection processes require further investigations, and again, multiarea multielectrode recordings in NHPs will shed light on systems‐wide oscillatory mechanisms underlying recognition memory. The fact that familiarity was not affected by beta rTMS over dlPFC in Experiment 2 is in line with human neuroimaging meta‐analyses suggesting that activation peaks related to familiarity in PFC were more often located between the inferior and the middle frontal gyrus (i.e., medial PFC) than in dlPFC (Scalici et al., 2017). According to a beta‐circuit model, familiarity processes are modulated by thalamo‐cortical synchronization within the beta frequency range, involving entorhinal, parahippocampal and perirhinal cortical areas connected via the medial dorsal thalamic nucleus to PFC (Ketz et al., 2015). Indeed, the functional role of rhinal cortex, especially perirhinal cortex, has long been associated more with familiarity‐based recognition than recollection (Aggleton & Brown, 1999; Bowles et al., 2010; Norman & O'Reilly, 2003; Ranganath et al., 2004; Skinner & Fernandes, 2007). rTMS was a viable methodology in our study due to dlPFC being easily accessible on the lateral surface of the frontal lobes, but this method is not viable for targeting deep structures in the PFC or MTL. Again, future multiarea recording work in NHPs will elucidate these system‐wide circuits and interactions, and so further work on developing bridges between human and animal models will be crucial.

### 4.4 Other theories/models on the functional role of BBA in working memory

It is useful to consider alternative accounts from the extant BBA literature that could speak to the causal effects of our low‐beta stimulation on recall. We next consider whether rTMS interfered with maintenance in working memory. One theory as to the functional relevance of BBA in lateral PFC for working memory relates BBA to low‐frequency baseline attractor dynamics, a so‐called idling rhythm associated with nonspecific spiking activity; that activity is periodically punctuated by high‐frequency gamma bursts associated with informationally relevant spiking activity when encoding or decoding the stimuli (Lundqvist et al., 2011, 2016). This model specifically predicts theta/gamma power increases and alpha/beta power decreases with memory load. Although the NHP version of our task does not explicitly set out to tax memory load per se (it does not have multiple test items per trial), memory load may be considered reasonably high because the trial‐unique match trials analysed in Experiment 1 are embedded in sessions with many nonmatch trials with trial unique stimuli as well as many match and nonmatch trials with familiar stimuli too. Hence, keeping novel stimulus items in match trials distinct in memory from interference imposes memory load. Perhaps consistent with the model's prediction that BBA drops with memory load, BBA in both NHPs does eventually drop below pre‐sample appearance baseline power in the sample period especially with respect to low‐beta ~300‐ms post‐sample appearance (Figure 3a). To avoid ceiling effects, the human version of the task (Experiment 2) imposes sample lists prior to choice trials, and hence memory load in Experiment 2 is high. It is logical then to expect our periodic 12.5‐Hz rTMS pulse trains (which target four out of the 12 samples in each list) may impact upon attractor dynamics and sustained working memory. Our within‐block analyses in Experiment 2 are consistent with low‐beta rTMS selectively affecting maintenance of samples directly targeted by stimulation, but our between‐block analyses in Experiment 2 suggest some degree of general affect to the whole block too. Recent data from macaque PFC directly support the model: Neurons that carried working memory information were associated with gamma‐modulated sites and showed lowered BBA (and accordingly high gamma power) during stimulus presentation/encoding (Lundqvist et al., 2016). Our NHPs in Experiment 1 do not show this pattern as we see peak BBA during stimulus presentation/encoding. That said, our data are not necessarily inconsistent with the model as we note the majority of sites recorded from in Lundqvist et al. (2016) were reported to *not* be gamma modulated and as a population showed BBA throughout the trial including stimulus presentation epochs. There are of course some significant differences between these studies; for example, our monkeys in Experiment 1 were not required to fixate during sample presentation that might conceivably relate to persistent versus transient BBA.

One further theory as to the functional relevance of BBA in PFC is that rather than reflecting baseline ‘idling’, BBA is instead a signature of ‘active’ maintenance of the status quo (Engel & Fries, 2010). According to this account, heightened BBA is associated with active maintenance of cognitive set, such as in tasks involving endogenous control as opposed to those driven by exogenous control. By this account, heightened BBA is also associated with active resistance from distraction, whereas lower BBA is associated instead with exogenous distracting inputs such as novel or unexpected events. Evidence in support of this includes monkeys trained to detect targets either by pop‐out or serial search that showed stronger frontal–parietal beta coupling during top‐down search but stronger gamma coupling in bottom‐up pop‐out attention (Buschman & Miller, 2007). Another study also indicated roles for low‐frequency oscillations in top‐down processing and higher frequency oscillations in bottom‐up processing (Kornblith et al., 2016). In our Experiment 1, we found transiently heightened BBA shortly after sample presentation. It could be argued, in line with the aforementioned theory, that endogenous control is required to encode any novel stimulus as a distinct new stimulus without interference (from all the familiar stimuli, see earlier). However, once adequately encoded, no other stimuli will appear until the choice test, and so maintenance demands may be low until that point, reflected in a sustained subsequent lowering of BBA. Although we cannot rule out such a post hoc explanation for our NHP BBA observations, it is not necessarily the most parsimonious account. For example, one might have instead hypothesized lower BBA during encoding if one considered a bottom‐up effect in processing novel stimuli according to this theory. That said, this theory would predict that, across multiple samples in lists in our human version of task, BBA would increase with memory load as participants proceeded through the sample list. Accordingly, one may speculate that periodic beta rTMS during the sample list may disrupt such active maintenance of working memory.

The heightened BBA during sample presentation might be explained by one further notion: It has been argued that increased BBA at the end of working memory trial might act as a ‘clear‐out’ signal when information needs to be erased. This has been likened to a BBA clear‐out function observed during stopping of actions and stopping of long‐term memory retrieval (stopping thoughts) in other brain regions (Schmidt et al., 2019). However, in our Experiment 1 task, the heightened BBA is observed at the start of the trial. Yet, this could in fact be the optimum place to locate a clear‐out signal in our NHP task; with only one relevant stimulus to ever remember at once (the single sample stimulus in that trial), a clear‐out of dlPFC‐mediated working memory at the point the next sample stimulus is expected or initially detected as being presented could optimally ensure only it was encoded without interference from anything else in such a ‘cleared‐out’ working memory.

### 4.5 Limitations of current study

We recognize a number of limitations inherent in Experiments 1 and 2. One issue is that although we find low‐beta rTMS during sample presentation affects subsequent remembrance of those samples, we do not know if the effect of stimulation is deleterious to attention, perception or mnemonic encoding of the sample. We have considered attentional and mnemonic hypotheses above but not yet perceptual accounts. In general, dlPFC is not strongly implicated in perception, but dlPFC interacts with posterior structures in the MTL and occipital lobe very implicated in perception so a perceptual role cannot be ruled out. The BBA power we observed in Experiment 1 appears very early after sample presentation (Figure 3) might lend itself to a perceptual account. However, on balance, we feel we can likely rule that out because a perceptual deficit is unlikely to selectively affect recollection leaving familiarity intact. It is true that recollection is associated with high confidence remembrance and a partial perceptual deficit might reduce confidence so leaving lower confidence familiarity judgements intact, but in Experiment 2, the beta stimulation leaves hit rate intact too and a perceptual deficit is unlikely to have produced such selective effects upon subsequent mnemonic processes. A second limitation is laterality; in Experiment 1, we only recorded from left frontal cortex in NHPs, and in Experiment 2, we only targeted low‐beta rTMS to left frontal cortex in humans. We cannot therefore generalize our results to the right frontal cortex. Much debate has already been had in the frontal cortex neuroimaging literature about laterality, primarily encoding/retrieval asymmetries versus stimulus‐domain‐specific functional lateralization (e.g., Habib et al., 2003). However, a comparative neuroimaging and neuropsychological patient testing study concluded that the memory‐related asymmetries observed during functional neuroimaging studies may not be critical for task performance as both left and right unilaterally lesioned patients showed similar deficits (Lee et al., 2002). With respect to laterality of rTMS per se, a previous study found that mid‐beta frequency rTMS (at 20 Hz) to either right or left dlPFC (albeit ~1 cm more rostral to our target) during sample stimulus encoding (in this case the discriminanda were faces) reduced subsequent familiarity estimates, but only right rTMS reduced recollection estimates (Turriziani et al., 2008). The differences in stimulus material, location, frequency and task all suggest further work is required and it would be unwise to generalize across hemispheres in the absence of those studies. A third issue is frequency specificity of effect. We found that low‐beta rTMS to left dlPFC reduced recollection, and we employed a very suitable control in one respect, namely, our random frequency stimulation that controlled for total number of pulses and total duration of the pulse train. We did not include any other rhythmic controls (e.g., rTMS at higher or lower frequencies than 12.5 Hz). Had we done so, we would of course have had to also include its matched randomly timed stimulation condition (i.e., with the same pulse number and same total duration). However, this would have made our Experiment 2 study unwieldy in the within‐subject and research project student context in which it was conducted (see Section 2). As stated clearly, our main aim in Experiment 2 was to test hypotheses of causality of rTMS protocols directly inspired by the LFP recordings in Experiment 1, and as such our study is only a preliminary investigation in this wider context. Although our own results from Experiment 1 in this particular task gave us no reason to think rTMS at other frequencies would be functionally relevant, our consideration of dynamic models of working memory and communication by coherence do suggest ways in which higher frequencies (high beta, gamma) or lower frequencies (alpha, theta) may enhance or interfere with working memory processes in an extended network of interconnected brain regions. Only a few previous studies have considered other frequencies of rTMS with respect to recollection and familiarity in frontal cortex per se. For example, 1‐Hz rTMS to left or right dlPFC at encoding significantly affected both recollection and familiarity of pictures of scenes (Turriziani et al., 2010). Hanslmayr et al. (2014) included examination of rTMS at 6.8 Hz, but encoding was selectively impaired only when BA9 was stimulated at a higher beta frequency (18.7 Hz); moreover, they did not include any random frequency control, nor did they examine recollection versus familiarity. More studies are clearly warranted as no study can possibly and reasonably include every relevant frequency and every relevant control. A fourth limitation is that in our laboratory, we did not have EEG available in our human participants so we could not check for echo entrainment in the manner of Hanslmayr et al. (2014). Similarly, we do not have rTMS available in our NHP laboratory so could not check the effects of rTMS upon LFPs (e.g., to verify rTMS induced echo in NHPs too). In any case, that would not be our advised or desired future direction in NHP systems neuroscience work aimed at building bridges between primate species; rather, we would prefer to use more spatially targeted interventions in NHPs that befit and complement the high spatial resolution of our recordings. Indeed, it is these higher resolution NHP methodologies compared with EEG/fMRI/rTMS in humans that are most likely to advance mechanistic neuronal level understanding. Yet, bridging the species divide is still an important goal to aspire to even if different methodologies are more appropriately employed in different species. A fifth limitation is the performance difference of the two NHPs. Animal B, in the sessions, we recorded, expressed a very low error rate compared with Animal A (Figure 3). However, both animals were suitably well trained on the task (despite more errors, Animal A still achieved ~75% correct). This is also why we plot data from each NHP separately and transparently (Figure 3) and do not simply average data across animals in these figures. The minor performance difference is not a major concern to us as both of the hit versus miss effects we report are apparent in both NHPs nonetheless. Moreover, even if we only considered correct response LFPs (and ignored errors in both NHPs) to determine the suggested rTMS frequency for Experiment 2, the 10‐ to 15‐Hz range would still have been the most consistent task‐relevant frequency, and 12.5‐Hz rTMS would still have been selected for Experiment 2.

To sum up, we hope the current project, despite limitations, will further inspire attempts to progress in understanding the systems‐wide neural mechanisms supporting primate object recognition memory. Many homologies exist between NHP and human PFC evidenced by long‐standing comparative cytoarchitectural analyses (e.g., Petrides & Pandya, 2007) and more recent comparative functional connectivity profiles (Neubert et al., 2015). We infer that there will be many shared mechanisms discovered between macaques and humans given similarities in primate brain organization. The current study at least serves as proof of principle that spatial and temporal parameters from NHP electrophysiological investigation can lead to discoveries in humans, and we expect vice versa to hold too. Lesion studies in macaques certainly point to extended circuits having causal relevance; for example, ventral PFC when lesioned results in a greater degree of impairment in object recognition memory than dorsal PFC (Bachevalier & Mishkin, 1986; Kowalska et al., 1991; Levy & Goldman‐Rakic, 1999; Meunier et al., 1997; Mishkin & Manning, 1978). The proposed functions of BBA in recollection and familiarity processes across a wider range of brain regions and stimulus domains need to be explored in future studies using a wider range of methodologies. There may not be any single functional description of BBA in PFC as all the above considerations taken together suggest. But we certainly expect further insights to arise from animal models wherein simultaneous multiarea multielectrode neuronal‐level recordings are combined with highly targeted intervention methodologies including reversible inactivations (e.g., direct pharmacological or opto‐/chemo‐genetic manipulations or direct microstimulation through the same implanted recording electrodes) to reveal causal influences within such extended circuits. For this to succeed, we need to develop tasks that can bridge the species divide.

## CONFLICT OF INTEREST

The authors declare no competing financial interests.

## AUTHOR CONTRIBUTIONS

Zhemeng Wu was responsible for the conceptualization, resources, experimentation, analyses and preparing manuscript. Martina Kavanova was responsible for the experimentation and preliminary analyses. Lydia Hickman was responsible for the experimentation and preliminary analyses. Erica A. Boschin was responsible for the resources, experimentation and supervision. Juan M. Galeazzi was responsible for the resources, experimentation and supervision. Lennart Verhagen was responsible for the resources and supervision. Matthew Ainsworth was responsible for the resources, experimentation and analyses. Carlos Pedreira was responsible for the funding acquisition (equipment grant) and resources. Mark J. Buckley was responsible for the funding acquisition (research grants and equipment grant), conceptualization, resources, experimentation, supervision, analyses and preparing the manuscript.

### PEER REVIEW

The peer review history for this article is available at https://publons.com/publon/10.1111/ejn.15535.

## Data Availability

All data and code are available upon request.
